# Ideal Time to Conduct a Pharmacokinetic Investigation After Delivery to Fully Capture the Effect of Pregnancy on Drug Exposure

**DOI:** 10.1093/ofid/ofae585

**Published:** 2024-10-15

**Authors:** Mattia Berton, Felix Stader, Sara Bettonte, Manuel Battegay, Catia Marzolini

**Affiliations:** Division of Infectious Diseases and Hospital Epidemiology, Departments of Medicine and Clinical Research, University Hospital Basel, Basel, Switzerland; Faculty of Medicine, University of Basel, Basel, Switzerland; Certara UK Limited, Sheffield, United Kingdom; Division of Infectious Diseases and Hospital Epidemiology, Departments of Medicine and Clinical Research, University Hospital Basel, Basel, Switzerland; Faculty of Medicine, University of Basel, Basel, Switzerland; Division of Infectious Diseases and Hospital Epidemiology, Departments of Medicine and Clinical Research, University Hospital Basel, Basel, Switzerland; Faculty of Medicine, University of Basel, Basel, Switzerland; Division of Infectious Diseases and Hospital Epidemiology, Departments of Medicine and Clinical Research, University Hospital Basel, Basel, Switzerland; Faculty of Medicine, University of Basel, Basel, Switzerland; Service of Clinical Pharmacology, Department of Laboratory Medicine and Pathology, University Hospital Lausanne and University of Lausanne, Lausanne, Switzerland; Department of Molecular and Clinical Pharmacology, University of Liverpool, Liverpool, United Kingdom

**Keywords:** antiretrovirals, HIV, pharmacokinetics, postpartum, pregnancy

## Abstract

**Background:**

The World Health Organization is pushing to accelerate the study of new human immunodeficiency virus drugs in pregnant women. However, regulatory guidelines do not specify when to conduct pharmacokinetic studies in postpartum women. This knowledge gap carries the potential to jeopardize the outcomes and conclusions of clinical trials aiming to study the effect of pregnancy on drug exposure. We used physiologically based pharmacokinetic (PBPK) modeling along with clinical data to determine the time needed after delivery for drug exposure to return to prepregnancy levels.

**Methods:**

A literature review was conducted to collect physiological parameters of pregnant and postpartum women. Regression analyses were performed to derive equations describing the parameters trajectory throughout pregnancy and post partum to inform our PBPK model. Published pharmacokinetic data in pregnant and postpartum women were used for the model verification. The PBPK model was subsequently applied to investigate pharmacokinetic changes throughout pregnancy and post partum.

**Results:**

In agreement with the clinical data the PBPK model was able to describe the different effects of pregnancy on drug exposure, with bictegravir showing the largest reduction in exposure (approximately 50%) during the third trimester while ritonavir and raltegravir showing the lowest (approximately 30%). The successfully verified PBPK model predicted that all evaluated antiretrovirals mostly return to prepregnancy exposure 4 weeks after delivery.

**Conclusions:**

Pharmacokinetic investigations on hepatically cleared drugs should not be conducted before the fifth week after delivery to fully characterize the effect of pregnancy on drug exposure. Because physiological changes remain after delivery, early measurements can underestimate the pregnancy effect on pharmacokinetics, leading to suboptimal dosing recommendations during pregnancy.

Traditionally, pregnant women have been excluded from clinical trials, but in recent years several regulatory documents have been issued to support the of conduct clinical investigations during pregnancy [1–3]. In addition, the World Health Organization has launched a call to accelerate the study of new human immunodeficiency virus (HIV) drugs in pregnant women, so that no woman is excluded from access to innovative drugs for the treatment and prevention of HIV infection [4].

Historically, clinical trials were not performed in order to prevent eventual drug-related adverse effects on the mother and unborn child. However, it is now commonly recognized that the lack of data in pregnancy is problematic, as it may prevent the use of a newer, potentially more effective treatment due to uncertainties in relation to safety questions and optimal dosing in this vulnerable population [5]. During the gestational period, the female body goes indeed through a series of anatomic, physiological, and biological changes [5, 6] that affect the pharmacokinetics/pharmacodynamics of the drug, leading possibly to lower exposure and the related loss of effectiveness.

Due to the necessity to prevent perinatal HIV transmission by ensuring sufficient drug exposure, many studies conducted to date in pregnant women are for antiretroviral drugs [7]. In these studies, pregnant women were generally followed up longitudinally, and full pharmacokinetic profiles were measured during the second and third trimesters as well as post partum in order to evaluate the impact of pregnancy on drug exposure. The women served as their own controls, and the effect of pregnancy was quantified by comparing the pharmacokinetic parameters in the second or third trimesters relative to post partum. While the time windows delimiting the second and the third trimesters are well defined, this is not the case for the postpartum period, so that pharmacokinetic investigations have been performed at variable times ranging from 2 to 40 weeks after delivery.

The lack of clear guidance on when to measure drug pharmacokinetics after delivery represents an important gap when characterizing the effect of pregnancy on drug exposure. Physiological changes in pregnant women peak at the third trimester; therefore, if pharmacokinetic investigations are done too early after delivery, there is a risk of not fully capturing the effect of pregnancy on drug exposure. Pregnancy-related physiological changes may indeed still be present, so that the difference in drug exposure between the second or third trimester and post partum will be mitigated.

Physiologically based pharmacokinetic (PBPK) modeling is a mathematical tool that considers both the physiological parameters of the population of interest and the drug physicochemical properties to predict concentration-time profiles and pharmacokinetic parameters [8]. It has successfully been applied to explore unstudied clinical scenarios in populations whose physiology differs from that of an healthy adult population [9–11]. The aim of the current study was to use PBPK modeling combined with observed clinical data for antiretroviral drugs to characterize pharmacokinetic changes during pregnancy and the postpartum period in order to provide guidance on when to conduct pharmacokinetic investigations in postpartum women.

## METHODS

We took 3 steps to investigate the time to return to prepregnancy drug exposure after delivery. First, we developed and implemented the pregnant and postpartum population in our in-house PBPK model [8]. Second, we used the available pharmacokinetic studies for antiretroviral drugs to verify the model ability to predict the pharmacokinetics during the second and third trimesters of pregnancy and during the postpartum period. Finally, we applied the PBPK model to simulate antiretroviral drug exposure during pregnancy and at different intervals (weeks) after delivery to determine the time window to return to prepregnancy drug metabolism.

### Pregnant and Postpartum Population Development

A detailed description of the physiological changes during pregnancy (gestational age 0–40 weeks) and the postpartum period (gestational age >40 weeks) is necessary to inform the PBPK model and simulate drug pharmacokinetics in pregnant and postpartum women. Existing pregnant and postpartum populations [12–15] were taken as a starting point, and a literature search using both PubMed and Google Scholar was performed to include newer studies investigating the anatomic, physiological, and biological (eg, enzyme and plasma protein abundances) changes during pregnancy.

**Figure 1.**
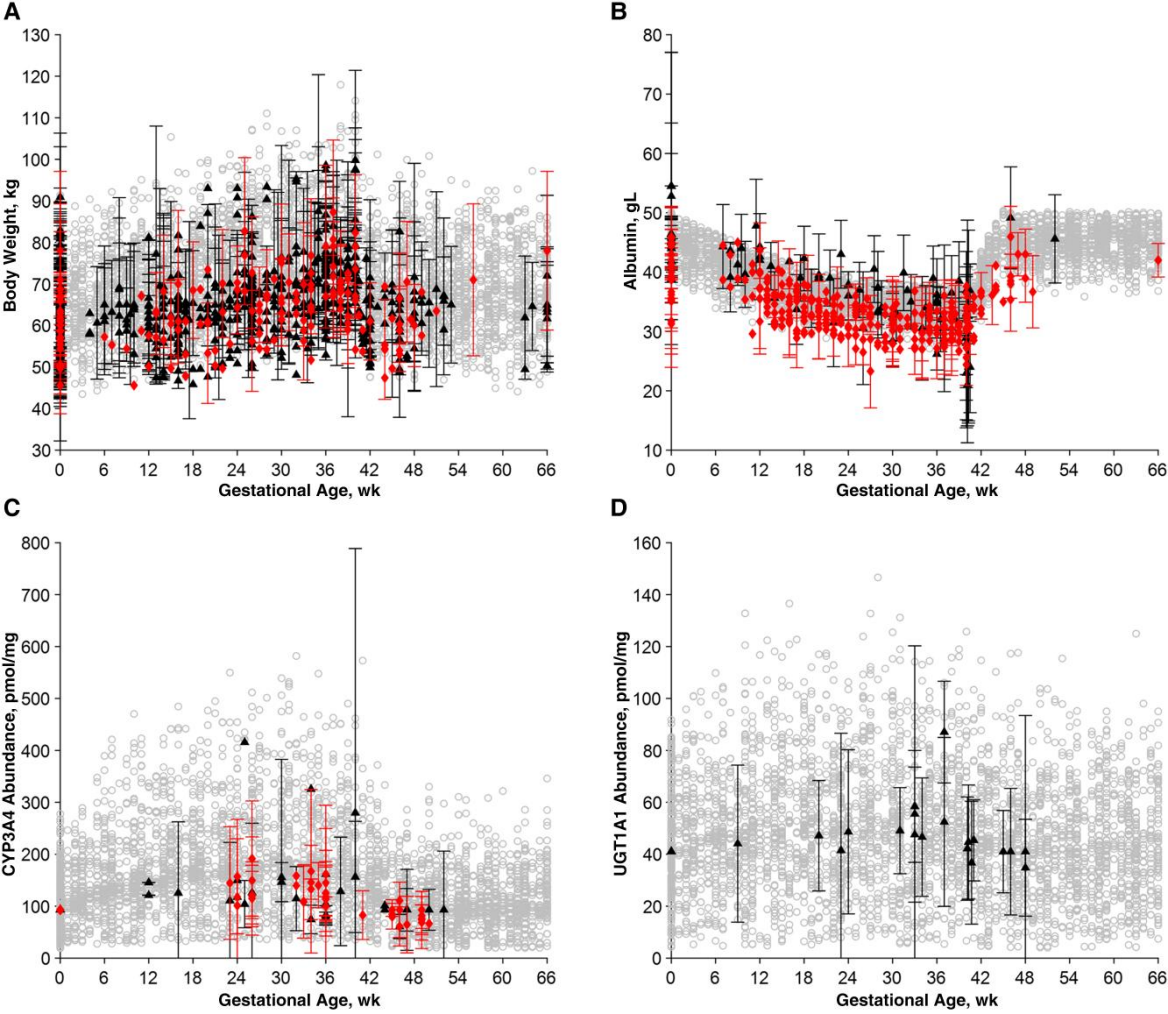
Body weight (*A*), albumin (*B*), cytochrome (CYP) 3A4 abundance (*C*), and UGT1A1 abundance (*D*) versus gestational age. Black triangles represent observed data from the development data set; red diamonds, data from the verification data set; and gray circles, predicted data points calculated using the derived equations. Data points with multiple women are represented as means with SDs.

The collected data were divided into development and verification data sets based on the number of covariates present in the study (Supplementary Table 1). The parameter values were then normalized to the prepregnancy values to derive the pharmacokinetic parameter fold change increase or decrease. A regression analysis was subsequently performed with Matlab 2020a software, using linear, polynomial, and exponential functions with gestational age as the only covariate. The best fitted function was selected based on visual and numerical diagnostics, such as the *R*^2^ value and the Akaike information criterion. The selected equations (Supplementary Table 2) were implemented in our in-house PBPK model developed in Matlab 2020a software [8], and the physiological parameters for 1000 women were generated across the gestational and postpartum periods from 0 to 66 weeks and compared against the verification data set (Figure 1 and Supplementary Figures 1–2).

### Pregnant PBPK Model Verification

A literature search was carried out to identify clinical studies in pregnant and postpartum women to verify the predictive performance of the developed PBPK model. Comprehensive clinical studies were found for 7 antiretroviral drugs (Supplementary Table 3). Information was collected about trial design, dosing regimen, participants’ demographic characteristics, and the pharmacokinetic results. If more than one study described the pharmacokinetics during the same gestational period, results were combined by calculating the weighted mean and standard deviation or geometric mean and coefficient of variance. The published concentration-time profiles were digitalized using GetData Graph Digitizer V.2.26 software. The drug PBPK models for the 7 evaluated antiretrovirals were developed previously and successfully applied to describe the pharmacokinetics in young, elderly, and obese people with HIV [10, 16]. Thus, they were used without changing any drug parameters to predict the pharmacokinetics in pregnant and postpartum women. Each simulation was performed by matching the participants’ characteristics reported in the clinical trial, such as age and gestational age range, as well as dose and dosing regimen (Supplementary Table 3). The pregnant and postpartum populations as well as the drug models were considered verified if the simulations were within 2-fold of observed data.

### Analysis of Antiretroviral Drug Exposure Across Different Postpartum Intervals

The verified PBPK models were subsequently used to simulate the antiretroviral exposure for the periods of interest, specifically, the third trimester of pregnancy and 2, 5, 6 7, 8, 12, and 16 weeks post partum. The pharmacokinetic results for peak concentration (C_max_), area under the curve (AUC) to time *t*, and C_τ_ were normalized to those obtained from the simulation in nonpregnant women to derive the fold change. The latter was related to the 1.25-fold bioequivalence interval, above which the fold change was considered clinically significant according to the US Food and Drug Administration and other regulatory bodies [17, 18].

## RESULTS

### Pregnant and Postpartum Population Development

A thorough literature search allowed us to identify 224 articles for the development of the pregnant and postpartum populations (Supplementary Table 1). Overall, the pregnancy period was better characterized than the postpartum period; nevertheless, the gathered data were sufficient to derive robust equations for all the physiological parameters of interest (Supplementary Table 2).

The body weight and the volume or weight of organs were mostly available (Figure 1*A* and Supplementary Figure 1*A* and 1*B*). These weights peaked during the third trimester and, compared with those in nonpregnant women; they were 25%, 30%, 30%, and 47% higher for the adipose tissue, heart, kidney, and blood and 7% lower for the brain. For the aforementioned organs, new equations were developed, while the equations for uterus and mammary gland weights were taken from Abduljalil and colleagues [12] and, based on what was described by Gaohua and colleagues [19], they were added to the muscle compartment. The equations for the weight of the fetus, placenta, and amniotic fluid were also taken from Abduljalil and colleagues [12], and these weights were then combined to calculate the total fetal-placental weight.

The change in cardiac output was well described in the literature and detailed description could be derived (Supplementary Figure 1*C*); it was found to be 20% higher during the third trimester compared with nonpregnant women, and it returned to baseline 10 weeks after delivery. On the other hand, finding tissue and organ blood flow data was more challenging, and only sparse information could be collected. For most organs, the blood flow was assumed to remain unchanged. Thus, given that the cardiac output increases throughout pregnancy, the percentage of cardiac output reaching the organ had to be adjusted accordingly. New equations were developed for blood flow in the brain, kidney, and uterus (Supplementary Table 2 and Figure 1*D* and 1*E*), and the fetal-placental blood flow was taken directly from Gaohua and colleagues [19].

Plasma protein levels were well described across the investigated gestational ages; both albumin and α-1 acid glycoprotein concentrations decreased throughout the pregnancy period (Figure 1*B* and Supplementary Figure 1*F*), reaching the lowest concentrations, 25% and 20%, respectively, during the third trimester (Table 1). Liver enzyme abundance data could not be found, as liver biopsy samples are generally not performed during pregnancy, so clinical data were used instead. A fair amount of clinical data was retrieved for both cytochrome (CYP) and uridine diphosphate (UDP)-glucuronosyltransferase (UGT) substrates (Supplementary Table 2). Fold differences in clearance between pregnant or postpartum women and nonpregnant women were calculated and used as a surrogate of enzyme abundance change. CYP and UGT enzymes were found to be induced during pregnancy, as shown in Figure 1*C* and 1*D* for CYP3A4 and UGT1A1, but CYP1A2 was found to be inhibited. The specific increase or decrease in expression for each enzyme is reported in Table 1, together with the estimated time needed for the enzyme expression to return to baseline.

**Table 1.** Changes in Enzyme Abundance and Albumin Concentration During Pregnancy and Time Needed for Return to Physiological Baseline

Variable	CYP1A2	CYP2B6	CYP2C9	CYP2C19	CYP2D6	CYP3A4	UGT1A1	UGT1A4	Albumin
Change between nonpregnant women and women at GA 37–40 wk, %	−44	30	56	24	44	69	23	116	−25
Time when parameter is back to baseline, postpartum wk	12	11	2	4	11	5	6	12	5

Abbreviations: CYP, cytochrome; GA, gestational age; UDP, uridine diphosphate; UGT, UDP glucuronosyltransferase.

### Pregnant and Postpartum Population Verification Against Clinical Observed Data

After implementing the pregnant and postpartum physiology in the PBPK model, we used clinical data from the literature to verify the ability of the model to describe the pharmacokinetics in this population.

#### Protease Inhibitors

We simulated the impact of pregnancy of ritonavir at 100 mg once daily. As ritonavir is used to boost protease inhibitors (Supplementary Figure 3), changes in ritonavir will also reflect changes in the boosted protease inhibitor. Ritonavir is a CYP3A4 substrate and, as supported by clinical data, its exposure is reduced by approximately 50% during the second and third trimesters. This reduction was well captured by the model (prediction was within the 1.25-fold error margin). The model also correctly predicted the exposure of ritonavir 6–12 weeks after delivery (within the 1.5-fold error margin; Table 2).

**Table 2.** Observed and Predicted Pharmacokinetic Parameters for Various Antiretrovirals in Nonpregnant, Pregnant, and Postpartum Women

Drug	Parameter	2nd Trimester			3rd Trimester			PP			2nd Trimester/PP Ratio			3rd Trimester/PP Ratio		
		Obs GM	Pred GM	Pred /Obs	Obs GM	Pred GM	Pred/Obs	Obs GM	Pred GM	Pred/Obs	Obs	Pred	Pred/Obs	Obs	Pred	Pred/Obs
Protease inhibitors																
GA, range, wk		24–28		…	34–38		…	46–52		…	…	…	…	…	…	…
Ritonavir (100 mg QD)^a^	C_max_, ng/mL	419	437	1.04	377	411	1.09	729	619	0.85	0.57	0.71	1.25	0.52	0.66	1.27
	AUC, ng ⋅ h/mL	3692	4336	1.17	3601	4874	1.35	6566	7158	1.09	0.56	0.61	1.09	0.55	0.68	1.24
	C_min_, ng/mL	24	33	1.38	38.4	58	1.51	52.8	76	1.44	0.45	0.43	0.96	0.73	0.76	1.04
Nonnucleoside reverse-transcriptase inhibitors																
GA, range, wk		14–28		…	28–39		…	42–54		…	…	…	…	…	…	…
Efavirenz (600 mg QD; EM/PM)	C_max_, ng/mL	3870	3129	0.81	5135	2905	0.57	4722	4310	0.91	0.82	0.73	0.89	1.09	0.67	0.61
	AUC, ng ⋅ h/mL	47 300	42 877	0.91	58 243	39 067	0.67	61 058	59 161	0.97	0.77	0.72	0.94	0.95	0.66	0.69
	C_min_, ng/mL	996	1353	1.36	1525	1205	0.79	1893	1910	1.01	0.53	0.71	1.34	0.81	0.63	0.78
GA, range, wk		…		…	13–40		…	41–80		…	…	…	…	…	…	…
Efavirenz (600 mg QD; EM)	C_max_, ng/mL	…	…	…	2640	2800	1.06	3190	4134	1.3	…	…	…	0.83	0.68	0.82
	AUC, ng ⋅ h/mL	…	…	…	25 900	37 361	1.44	52 400	54 494	1.04	…	…	…	0.49	0.69	1.41
	C_min_, ng/mL	…	…	…	1000	1139	1.14	2030	1745	0.86	…	…	…	0.49	0.65	1.33
GA, range, wk		24–28		…	30–38		…	43–54		…	…	…	…	…	…	…
Rilpivirine (25 mg QD)	C_max_, ng/mL	89	112	1.26	86	113	1.31	120	183	1.53	0.74	0.61	0.82	0.72	0.62	0.86
	AUC, ng ⋅ h/mL	1888	1898	1.01	1730	1958	1.13	2703	3204	1.19	0.7	0.59	0.84	0.64	0.61	0.95
	C_min_, ng/mL	59.05	54	0.91	54	56	1.04	87	99	1.14	0.68	0.55	0.81	0.62	0.57	0.92
GA, range, wk		13–27		…	28–40		…	46–52		…	…	…	…	…	…	…
Doravirine (100 mg QD)	C_max_, ng/mL	…	777	…	…	668	…	…	1228	…	…	0.63	…	…	0.54	…
	AUC, ng ⋅ h/mL	…	9587	…	…	8610	…	…	14 484	…	…	0.66	…	…	0.59	…
	C_min_, ng/mL	…	170	…	…	175	…	…	249	…	…	0.68	…	…	0.7	…
Integrase inhibitors																
GA, range, wk		20–26		…	27–38		…	43–64		…	…	…	…	…	…	…
Dolutegravir (50 mg QD)	C_max_, ng/mL	3620	2415	0.67	3022	2248	0.74	4378	3524	0.8	0.83	0.69	0.83	0.69	0.64	0.93
	AUC, ng ⋅ h/mL	47 600	30 063	0.63	41 952	29 077	0.69	59 375	52 254	0.88	0.8	0.58	0.73	0.71	0.56	0.79
	C_min_, ng/mL	730	415	0.57	764	453	0.59	1202	1080	0.9	0.61	0.38	0.62	0.64	0.42	0.66
GA, range, wk		13–27		…	28–40		…	46–52		…	…	…	…	…	…	…
Bictegravir (50 mg QD)	C_max_, ng/mL	5239	3590	0.69	4682	3162	0.68	10 066	5984	0.59	0.52	0.6	1.15	0.47	0.53	1.13
	AUC, ng ⋅ h/mL	59 768	60 169	1.01	56 741	52 998	0.93	139 284	112 932	0.81	0.43	0.53	1.23	0.41	0.47	1.15
	C_min_, ng/mL	1076	1550	1.44	1045	1404	1.34	3543	3524	0.99	0.3	0.44	1.47	0.29	0.4	1.38
GA, range, wk		20–26		…	32–40		…	43–51		…	…	…	…	…	…	…
Raltegravir (400 mg BID)	C_max_, ng/mL	2250	1637	0.73	1655	1723	1.04	2625	2067	0.79	0.86	0.79	0.92	0.63	0.83	1.32
	AUC, ng ⋅ h/mL	6600	5557	0.84	5264	5986	1.14	10 157	7095	0.7	0.65	0.78	1.2	0.52	0.84	1.62
	C_min_, ng/mL	62	92	1.48	68	107	1.57	93	126	1.35	0.67	0.73	1.09	0.73	0.85	1.16

Abbreviations: AUC_t_, area under the curve to time *t*; BID, twice daily; C_max_, peak concentration; C_min_, minimum concentration; EM, extensive metabolizer; GA, gestational age; GM, geometric mean; Obs, observed; PM, poor metabolizer; PP, post partum; Pred, predicted; Pred/Obs, ratio of predicted to observed value; QD, once daily.

^a^Results reported as means; all other results are reported as GMs.

#### Nonnucleoside Reverse-Transcriptase Inhibitors

**Figure 2.**
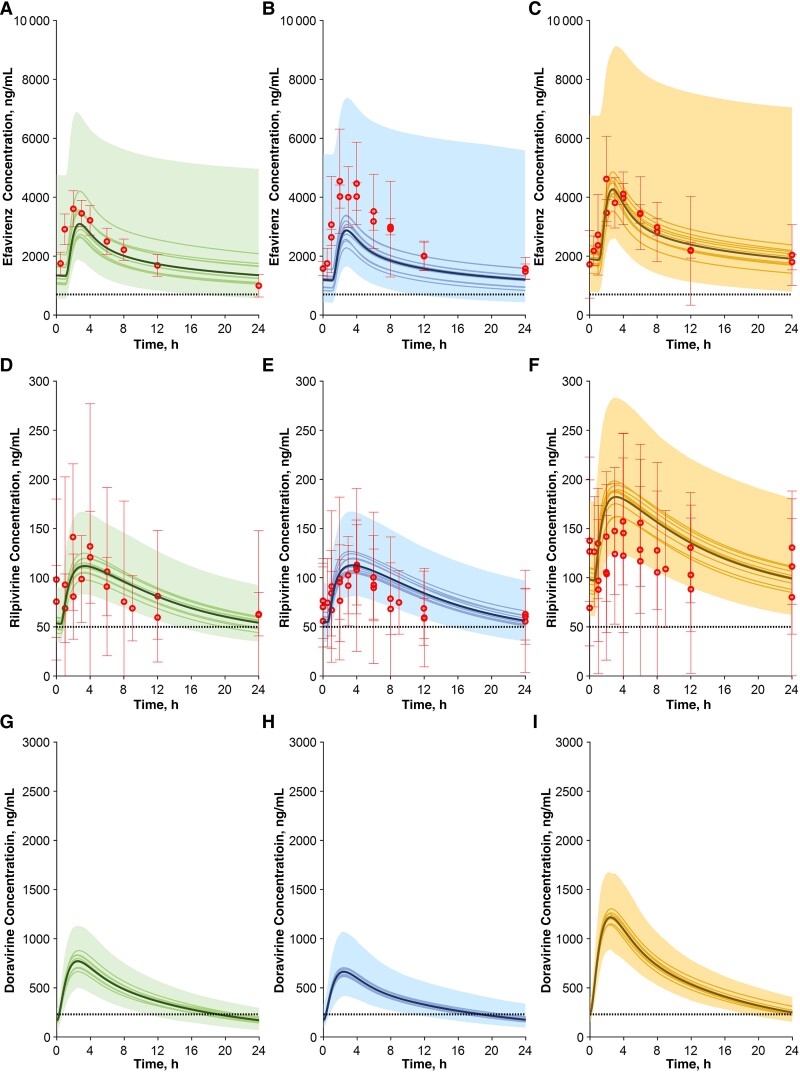
Concentration-time profile of efavirenz, rilpivirine, and doravirine in pregnant women during the second (*A*, *D*, *G*) and third (*B*, *E*, *H*) trimesters and in postpartum women (*C*, *F*, *I*). Red circles represent clinical observed data obtained from the literature; solid bold lines, solid lines, and shaded areas represent the simulated mean of all virtual trials, the mean of each virtual trial, and the 90% normal range, respectively; and dashed lines represent the clinical efficacy thresholds.

Three nonnucleoside reverse-transcriptase inhibitors were simulated, namely, efavirenz, rilpivirine, and doravirine. Efavirenz is a substrate of CYP2B6, which is subject to genetic polymorphisms. Three studies reported efavirenz exposure in pregnant and postpartum women (efavirenz dose, 600 mg once daily). Two of them did not specify the polymorphisms [20, 21] but reported that, based on the pharmacokinetic results, some participants likely carried genetic variations associated with a poor metabolism phenotype [20]. In the third study, various polymorphisms were studied; however, we considered only the data of rapid metabolizers, with the limitation that the exact gestational age was not reported [22]. Nevertheless, by matching the polymorphism characteristics of the study, we were able to obtain good simulations (Figure 2*A*–2*C*, Supplementary Figure 3), with the majority of results within the 1.5-fold error margin (Table 2).

Rilpivirine is a CYP3A4 substrate administered at an oral dose of 25 mg once daily. The model was able to predict (within the 1.25-fold error margin) the observed reduction (about 30%/40%) in both AUC and Cτ in the second and third trimesters (Figure 2*D*–2*F* and Table 2). Clinical data were not available for doravirine, except in a PBPK modeling study, which reported reductions of doravirine AUC and minimum concentration (C_min_) by 46% and 75%, respectively, during the second trimester and by 55% and 84% during the third [23]. Our model predicted a similar decrease for AUC (by 40%) for both trimesters, but C_min_ was predicted to be reduced by only 30% (Figure 2*G*–2*I* and Table 2). Regardless, both this and the published study predicted doravirine Cτ concentrations to be below the 230 ng/mL target trough concentration. Furthermore, the analysis we report in Table 3 clearly shows that during the third trimester a much higher percentage of women fall below the clinical target threshold (85% vs 29% in nonpregnant women).

**Table 3.** Percentages of Nonpregnant, Pregnant, and Postpartum Virtual Individuals With Predicted Plasma Concentrations Below the Clinical Target Trough Threshold

Comparison Group	Virtual Individuals With Predicted Plasma Concentration Below Clinical Target Trough Threshold, %^a^					
	Efavirenz	Rilpivirine	Doravirine	Dolutegravir	Bictegravir	Raltegravir
Nonpregnant individuals	3	0	29	1	3	0
Pregnant and postpartum individuals						
3rd Trimester	23	38	85	25	21	0
Postpartum wk						
2	19	21	67	16	11	0
5	6	1	49	8	1	0
6	7	1	44	1	0	0
7	8	2	50	6	2	0
8	3	0	46	5	1	0
12	3	0	37	3	0	0
16	3	1	38	1	1	0

^a^The target thresholds were 700 ng/mL for efavirenz [24], 50 ng/mL for rilpivirine [25], 230 ng/mL for doravirine [26], 300 ng/mL for dolutegravir [27], 760 ng/mL for bictegravir [28], and 20 ng/mL for raltegravir [29].

#### Integrase Inhibitors

**Figure 3.**
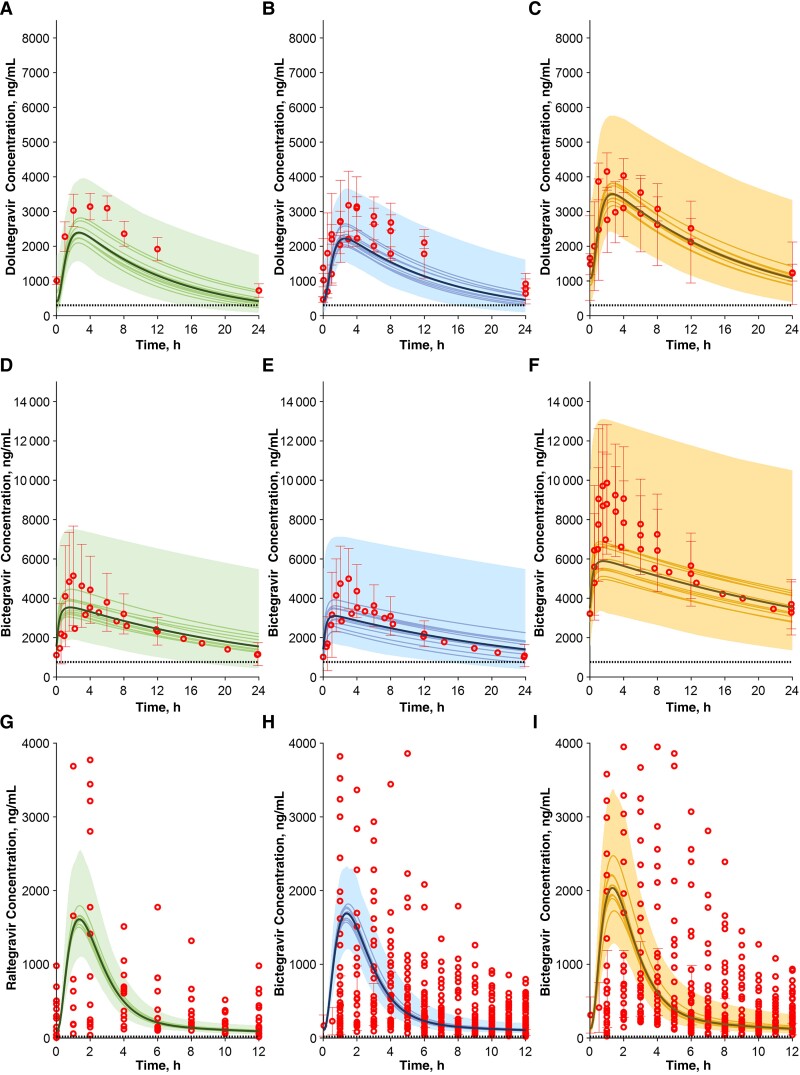
Concentration-time profile of dolutegravir, bictegravir, and raltegravir in pregnant women during the second (*A*, *D*, *G*) and third (*B*, *E*, *H*) trimesters and in postpartum women (*C*, *F*, *I*). Red circles represent clinical observed data obtained from the literature; solid bold lines, solid lines, and shaded areas represent the simulated mean of all virtual trials, the mean of each virtual trial, and the 90% normal range, respectively; and dashed lines represent the clinical efficacy thresholds.

Clinical studies were available for dolutegravir (50 mg once daily), bictegravir (50 mg once daily), and raltegravir (400 mg twice daily). Dolutegravir is mainly metabolized by UGT1A1 and to a lesser extent by CYP3A4 and therefore is subject to reduction in exposure during pregnancy. The model simulations predicted a 30% decrease in exposure during pregnancy compared with post partum, which agrees with observed data (predictions were within the 1.5-fold error margin) (Figure 3*A*–3*C* and Table 2). Bictegravir is equally metabolized by CYP3A4 and UGT1A1. The model predicted reductions of 50% and 70% in AUC and Cτ, respectively (predictions were within the 1.25-fold error margin) (Figure 3*D*–3*F* and Table 2). Finally, raltegravir is a pure UGT1A1 substrate, and its exposure was predicted to be reduced in agreement with observed data, even if the population variability was not fully captured by the model (Figure 3*G*–3*I* and Table 2). However, large variability for raltegravir has also been reported in other populations and has been attributed to variable absorption.

#### Summary

Overall, the model predicted 53%, 89%, and 100% of the pharmacokinetic parameters within the 1.25-, 1.5-, and 2-fold error margins, respectively. None of the predictions fell outside the 2-fold error margin, thereby qualifying the PBPK model for the prediction of the pharmacokinetics in both pregnant and postpartum populations.

### Pharmacokinetic Changes at Various Weeks Post Partum

**Figure 4.**
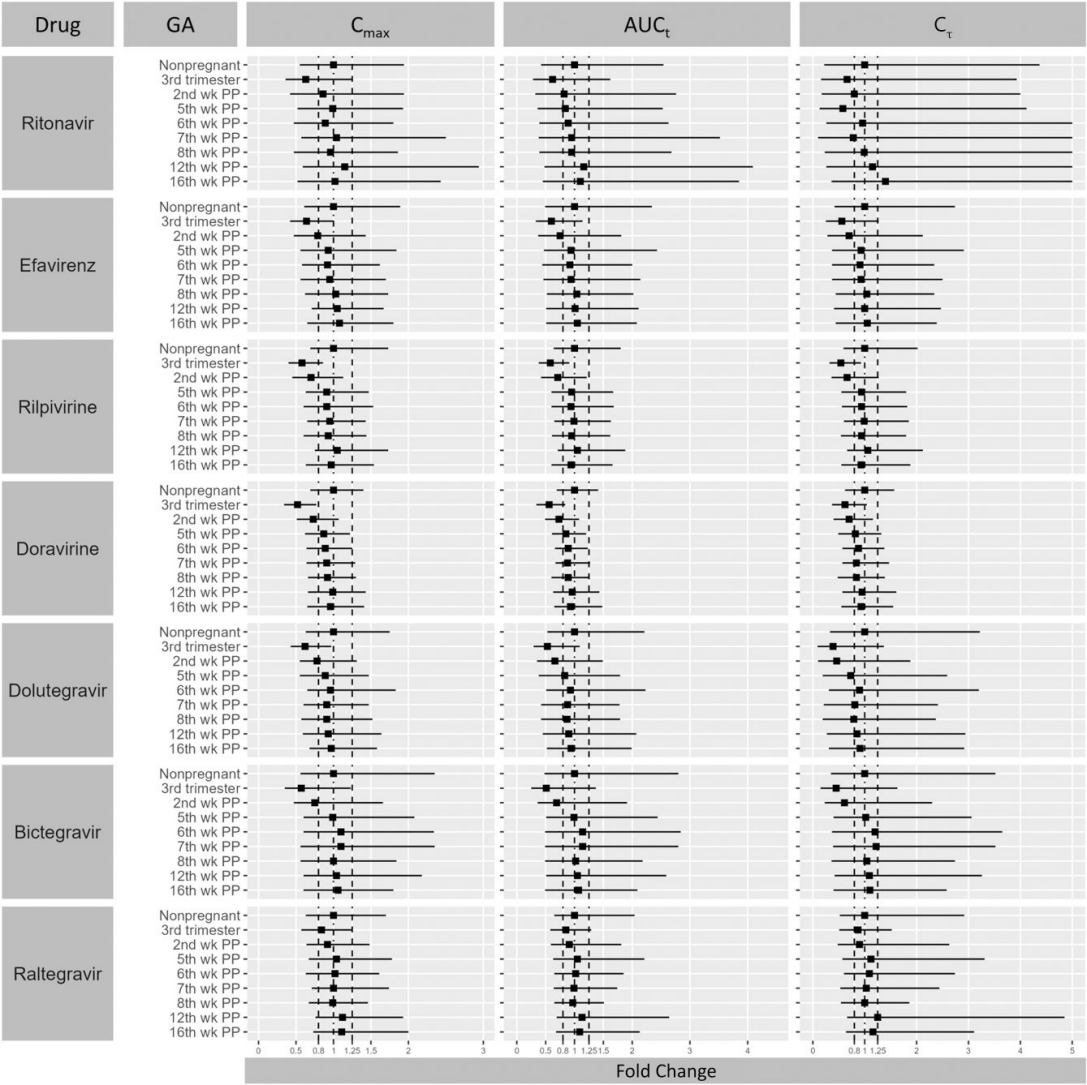
Predicted pharmacokinetic parameters fold changes across different gestational weeks. Data are expressed as geometric mean and fifth and 95th percentiles. Abbreviations: AUC_t_, area under the curve to time *t*; C_max_, peak concentration; C_τ_, trough concentration; GA, gestational age; PP, post partum.

The validated PBPK model was applied to investigate the pharmacokinetic changes at various weeks post partum and to determine the time window for returning to prepregnancy metabolism after delivery. Our simulations showed that C_max_, AUC, and Cτ are affected differently by the physiological changes during pregnancy and post partum (Figure 4). Specifically, in the third trimester the exposures of ritonavir and raltegravir were predicted to be the least affected (approximately 30%) while bictegravir showed the highest decrease (approximately 50%). Furthermore, the time needed for the antiretroviral drugs to go back to their prepregnancy pharmacokinetic profiles was variable, with most drugs requiring 5–6 weeks, or possibly 12 weeks for doravirine.

However, if we factor in the population variability that is commonly considered acceptable (ie, the 1.25-fold bioequivalence range), we can conclude that all pharmacokinetic parameters are within this range by week 5 (Figure 4). We also calculated the percentage of virtual women at various weeks post partum with plasma concentrations below the clinical target threshold. From Table 3, it can be seen how this percentage is high for most antiretrovirals during the third trimester and the second week post partum due to lower C_min_ concentrations; at the fifth week, however, this percentage is similar to that in nonpregnant women. Therefore, our findings suggest that a 4-week time window is needed after delivery before conducting pharmacokinetic investigations in order to capture the full effect of pregnancy on drug pharmacokinetics.

## DISCUSSION

There is a global effort to accelerate the study of new drugs by including more pregnant women in clinical trials so that this population can also have access to innovative drugs [1–3, 5, 7]. To fully characterize the effect of pregnancy on drug exposure, it is critical to determine the time window for drug metabolism to return to baseline.

To our knowledge, this is the first study providing a comprehensive analysis of antiretroviral drug pharmacokinetics in the postpartum period relative to the pharmacokinetics in nonpregnant women, using a PBPK model implemented with physiological changes in pregnancy and the postpartum period. The validated model showed differences in the time needed for antiretrovirals to return to baseline metabolism. Raltegravir and ritonavir exposures were predicted to already be comparable to those in nonpregnant women by 2 weeks after delivery. However, when considering all evaluated antiretrovirals, the pharmacokinetic parameters were mostly back to prepregnancy levels at week 5 after delivery. Thus, our work indicates that any pharmacokinetic investigation for which pregnant and postpartum women serve as their own control, a 4-week time window after delivery should be applied before sampling the pharmacokinetic curve. Since the effect of pregnancy on drug exposure is calculated by comparing the pharmacokinetic parameters in the second or third trimester with those in the postpartum period, if the investigation is conducted too early the physiological changes may still be present after delivery and lead to an underestimation of the effect of pregnancy.

In 2016 Colbers and colleagues [30] presented some data on the best time to conduct pharmacokinetic investigations in postpartum women and found that antiretroviral drug pharmacokinetics was back to baseline (ie, prepregnancy level) 3 weeks after delivery. However, this analysis had limitations, as antiretrovirals were not considered separately and only a few pharmacokinetic profiles were available in the postpartum period.

Our 4-week postdelivery time window before investigations is in line with the current practice as most pharmacokinetic studies are done 6–12 weeks after delivery. The need for a 4-week time window is due to the fact that some physiological changes persist after delivery. The liver enzymes are induced by estrogen and progesterone [31], hormones produced mainly by the placenta during pregnancy. After delivery, with removal of the placenta, the hormonal levels decline quickly, coming back to baseline values within a week [32]. Assuming that these 2 hormones are the only factors responsible for enzyme induction, one would assume that the metabolic enzymes should go back to baseline values within 2 weeks after delivery, as demonstrated in a modeling study [33].

However, it is important to highlight that the metabolism of a drug is not only influenced by the enzyme abundance but also by the plasma protein concentrations, which are lower during pregnancy. The albumin concentration is lower, notably due to the increase in blood volume, also called hemodilution. After delivery, the albumin concentration goes back to baseline only after 5 weeks post partum, which is in agreement with the time needed for the plasma and blood to go back their its own baseline volume, as also observed by Dallmann and colleagues [15]. Thus, taken together, these physiological changes support our 4-week time window before conducting pharmacokinetic investigations.

It is also important to pay attention when comparing postpartum data with data from nonpregnant adults or historical data because the pharmacokinetics are different in males compared with females, with female showing higher exposures at steady state [34]. Therefore, if the reference data do not distinguish sex or are issued from a predominantly male population, the reference data will likely show lower exposure than in postpartum women. Thus, we suggest systematic comparison of postpartum and pregnancy data against data from nonpregnant women to avoid any bias or misinterpretation. If only mixed-sex historical data are available, then it should be good practice to state the percentage of males or females.

The current work covers an important knowledge gap and brings several novelties, but we have to acknowledge some limitations, such as the sparse information on tissue and organ blood flow or the lack of enzyme abundance data. To overcome this latter issue, we have derived enzyme abundance data from clinical data in pregnant and postpartum versus nonpregnant women. The absorption was not mechanistically modeled, and a simple CAT model with effective permeability was used; this why C_max_ was not predicted as well as AUC and C_min_, but our focus was on the last 2 pharmacokinetics parameters. Furthermore, an assumption was made that all virtual simulated women delivered at exactly 40 weeks of gestation. In practice, however, this is not the case, with women delivering before and after 40 weeks; in future, it might be interesting to study whether delivering before or after the calculated due date influences the speed at which pregnancy-associated physiological changes will subside.

To summarize, the current study focused on the pharmacokinetics of antiretroviral drugs because there is particular interest in conducting clinical trials in pregnant and postpartum women with HIV to offer them the best treatment option and reduce the risk of perinatal transmission. However, the findings of this study can be generalized to other drugs, which are eliminated predominantly through hepatic metabolism via CYP3A4, 2B6, or UGT1A1 enzymes. These findings should not be applied to drugs eliminated renally, since this elimination route was not investigated, or to pregnant and postpartum women presenting with medical conditions that can affect drug pharmacokinetics.

In conclusion, to successfully design a clinical trial in pregnant and postpartum women, it is important to consider that the physiological changes related to pregnancy are still present in the first weeks after delivery, which may lead to an underestimation of the pregnancy effect on drug exposure and consequently to suboptimal dosing recommendations during pregnancy. Simulations using PBPK modeling combined with clinical data of several hepatically clear drugs indicate that a 4-week time window after delivery is needed before conducting pharmacokinetic investigations, in order to fully characterize the effect of pregnancy on drug pharmacokinetics.

## Supplementary Material

ofae585_Supplementary_Data

## Supplementary Data

Supplementary materials are available at *Open Forum Infectious Diseases* online. Consisting of data provided by the authors to benefit the reader, the posted materials are not copyedited and are the sole responsibility of the authors, so questions or comments should be addressed to the corresponding author.
