# Supplementary material for: Ideal Time to Conduct a Pharmacokinetic Investigation After Delivery to Fully Capture the Effect of Pregnancy on Drug Exposure
Source: Open Forum Infect Dis. 2024 Oct 15;11(10):ofae585. doi: 10.1093/ofid/ofae585 (PMC11495486; doi:10.1093/ofid/ofae585)
Supplement: ofae585_Supplementary_Data [file ofae585_supplementary_data.docx]

Supplementary Material

Title: Ideal time to conduct a pharmacokinetic investigation after delivery in order to fully capture the effect of pregnancy on drug exposure

Authors: Mattia Berton^1, 2^, Felix Stader^3^, Sara Bettonte^1, 2^, Manuel Battegay^1, 2^, Catia Marzolini^1, 2, 4, 5^

Affiliations: 1 Division of Infectious Diseases and Hospital Epidemiology, Departments of Medicine and Clinical Research, University Hospital Basel, 4031 Basel, Switzerland.

2 Faculty of Medicine, University of Basel, 4031 Basel, Switzerland.

3 Certara UK Limited, Sheffield, UK.

4 Service and Laboratory of Clinical Pharmacology, Department of Laboratory Medicine and Pathology, University Hospital Lausanne and University of Lausanne, Lausanne, Switzerland.

5 Department of Molecular and Clinical Pharmacology, University of Liverpool, United Kingdom.

Complete contact information for the corresponding authors:

Mattia Berton, MS

Division of Infectious Diseases and Hospital Epidemiology

Departments of Medicine and Clinical Research

University Hospital Basel

Petersgraben 4

4031 Basel, Switzerland

E-mail: mattia.berton@unibas.ch

*Alternative corresponding author:*

Catia Marzolini, PharmD, PhD

Division of Infectious Diseases and Hospital Epidemiology

Departments of Medicine and Clinical Research

University Hospital Basel

Petersgraben 4

4031 Basel, Switzerland

E-mail: catia.marzolini@usb.ch

Keywords: pregnancy, postpartum, pharmacokinetics, antiretrovirals, HIV

**Supplementary table 1**. List of articles used for the development and verification of the anatomical, physiological, and biological parameters fold change occurring during pregnancy and postpartum.

| **Parameter** | **References** |
| --- | --- |
| **Anthropometric parameters** | |
| Body weight FC | [1-92] |
| Adipose weight FC | [1, 2, 4, 5, 8-10, 17, 18, 20, 25, 27, 32, 34-36, 44, 53, 58-62, 75, 89, 90] |
| Brain weight FC | [93] |
| Heart weight FC | [11, 46, 50, 56, 57, 68, 72, 73, 94, 95] |
| Kidney weight FC | [96, 97] |
| Plasma weight FC | [4, 7, 67, 69, 74, 76, 79, 86, 88, 98-114] |
| Red blood cells weight FC | [4, 7, 67, 69, 76, 79, 88, 98, 99, 102, 105, 106, 109, 111, 113] |
| **Blood flow parameters** | |
| Cardiac output FC | [3, 4, 6, 7, 9, 11, 14-16, 18, 19, 22-24, 45-48, 50, 52, 56, 57, 68, 72, 73, 78, 95, 110, 115-126] |
| Brain blood flow FC | [127, 128] |
| Kidney blood flow FC | [4, 7, 99, 129-131] |
| Uterus blood flow FC | [132] |
| **Other model parameters** | |
| GFR FC | [4, 7, 23, 45, 64, 65, 71, 77, 83, 84, 87, 99, 103, 129-131, 133-142] |
| HCT FC | [3, 4, 7, 13, 35, 54, 67, 73-81, 83, 88, 99, 102, 106, 111, 121, 126, 129, 131, 143-146] |
| Albumin FC | [69, 70, 83, 87, 100, 101, 104, 114, 143, 144, 147-159] |
| AAG FC | [70, 143, 147-150, 160-163] |
| **Liver enzymes abundance** | |
| CYP1A2 FC | [83, 164-171] |
| CYP2B6 FC | [172-174] |
| CYP2C9 FC | [175, 176] |
| CYP2C19 FC | [177-184] |
| CYP2D6 FC | [171, 185-190] |
| CYP3A4 FC | [87, 150, 171, 175, 179, 181, 188, 191-205] |
| UGT1A1 FC | [206-211] |
| UGT1A4 FC | [212, 213] |

*AAG* alpha-1 acid glycoprotein, *FC* fold change, *GFR* glomerular filtration rate, *HCT* haematocrit.

**Supplementary table 2**. Equations describing anatomical, physiological, and biological parameters changes during pregnancy and postpartum, and necessary to inform a physiologically based pharmacokinetic

| Parameter | Pregnancy status | Gestational age (GA) | Descriptive equation | CV [%] | References |
| --- | --- | --- | --- | --- | --- |
| **Anthropometric parameters** | | | | | |
| Age | Non pregnant, pregnant, and postpartum |  | $Random Uniform Distribution$ |  |  |
| Body height [cm] | Non pregnant, pregnant, and postpartum | All GA | $-0.0039\times{Age}^{2}+0.238*Age-12.5 *Sex+176$ | 3.8 | [214] |
| Body weight [kg] | Non pregnant | - | $-0.0039\times{Age}^{2}+1.12\times Body height+0.611\times Age-0.424\times Sex-137$ | 15.2 | [214] |
| Body weight FC | Pregnant | <= 40 | $4.85E-05*GesAge^{2} + 4.05E-03*GesAge + 1$ |  |  |
| Body weight FC | Postpartum | > 40 | $3.38E-06*GesAge^{2}- 1.28E-03*GesAge + 1.11$ |  |  |
| Body mass index (BMI) [kg/m^2^] | Non pregnant, pregnant, and postpartum | All GA | $Body weight/({(Body height/100)}^{2})$ |  |  |
| Body surface area (BSA) [m^2^] | Non pregnant, pregnant, and postpartum | All GA | $0.007184*Body height^{0.725}*Body weight^{0.425}$ |  | [215] |
| **Organ weights [kg]** | | | | | |
| Lung weight | Non pregnant | - | $e^{\left( 0.028\times Body height+0.0077\times Age-5.6 \right)}$ | 0 | [214] |
| Lung weight FC | Pregnant and postpartum | All GA | $1$ |  |  |
| Adipose weight | Non pregnant | - | $0.68\times Body weight-0.56\times Body height+6.1\times Sex+65$ | 29.6 | [214] |
| Adipose weight FC | Pregnant | <= 40 | $6.75E-03*GesAge + 1$ |  |  |
| Adipose weight FC | Postpartum | > 40 | $-1.88E-03*GesAge + 1.24$ |  |  |
| Bone weight | Non pregnant | - | $e^{\left( 0.024\times Body height-1.9 \right)}$ | 13.2 | [214] |
| Bone weight FC | Pregnant and postpartum | All GA | $1$ |  |  |
| Brain weight | Non pregnant | - | $e^{-0.0075\times Age+0.0078\times Body height-0.97}$ | 9.0 | [214] |
| Brain weight FC | Pregnant | <= 40 | $-1.49E-03*GesAge + 1$ |  |  |
| Brain weight FC | Postpartum | > 40 | $1.22E-03*GesAge + 0.95$ |  |  |
| Brain weight FC | Postpartum | > 42 | $1$ |  |  |
| Gonad weight | Non pregnant | - | $-0.00034\times Body weight-0.00022\times Age-0.03\times Sex+0.072$ | 34.8 | [214] |
| Gonad weight FC | Pregnant and postpartum | All GA | 1 |  |  |
| Heart weight | Non pregnant | - | $0.34\times BSA+0.0018\times Age-0.36$ | 20.3 | [214] |
| Heart weight FC | Pregnant | <= 40 | $6.74E-03*GesAge + 1$ |  |  |
| Heart weight FC | Postpartum | > 40 | $-1.35E-03*GesAge + 1.12$ |  |  |
| Heart weight FC | Postpartum | > 86 | $1$ |  |  |
| Kidney weight | Non pregnant | - | $-0.00038\times Age-0.056\times Sex+0.33$ | 23.2 | [214] |
| Kidney weight FC | Pregnant | <= 40 | $7.32E-03*GesAge + 1$ |  |  |
| Kidney weight FC | Postpartum | > 40 | $-2.93E-02*GesAge + 2.46$ |  |  |
| Kidney weight FC | Postpartum | > 50 | $1$ |  |  |
| Uterus weight | Non pregnant | - | $0.079$ | 14.8 | [132] |
| Uterus weight FC | Pregnant | <= 40 | $2.74E-01*GesAge + 1$ |  | [132] |
| Uterus weight FC | Postpartum | > 40 | $71822*e^{\left( -0.225*GesAge \right)}$ |  | [132] |
| Uterus weight FC | Postpartum | > 49.7 | $1$ |  | [132] |
| Mammary gland | Non pregnant | - | $0.720$ | 51.65 | [132] |
| Mammary gland FC | Pregnant | <= 40 | $3.67E-02*GesAge + 1$ |  | [132] |
| Mammary gland FC | Postpartum | > 40 | $-1.29*GesAge + 54$ |  | [132] |
| Mammary gland FC | Postpartum | > 41.2 | $1$ |  | [132] |
| Muscle weight | Non pregnant | - | $17.9\times BSA-0.0667\times Age-5.68\times Sex-1.22$ | 11.8 | [214] |
| Muscle weight FC | Pregnant and postpartum | All GA | $1$ |  |  |
| Total muscle weight | Pregnant and postpartum | All GA | $\left( Muscle weight - Uterus weight - Mammary gland weight \right)* Muscle weight FC + Uterus weight * Uterus weight FC + Mammary gland weight * Mammary gland weight FC$ |  |  |
| Skin weight | Non pregnant | - | $e^{\left( -0.0058\times Age-0.37\times Sex+1.13 \right)}$ | 8.3 | [214] |
| Skin weight FC | Pregnant and postpartum | All GA | $1$ |  |  |
| Thymus weight | Non pregnant | - | $0.0221$ | 44.8 | [214] |
| Thymus weight FC | Pregnant and postpartum | All GA | $1$ |  |  |
| Gut weight | Non pregnant | - | $3E-06 \times{Body height}^{2.49}$ | 7.3 | [214] |
| Gut weight FC | Pregnant and postpartum | All GA | $1$ |  |  |
| Spleen weight | Non pregnant | - | $e^{1.13\times BSA-3.93}$ | 51.7 | [214] |
| Spleen weight FC | Pregnant and postpartum | All GA | $1$ |  |  |
| Pancreas weight | Non pregnant | - | $0.103$ | 27.8 | [214] |
| Pancreas weight FC | Pregnant and postpartum | All GA | $1$ |  |  |
| Liver weight | Non pregnant | - | $e^{(0.87\times BSA-0.0014\times Age-1.0)}$ | 23.7 | [214] |
| Liver weight FC | Pregnant and postpartum | All GA | $1$ |  |  |
| Blood weight | Non pregnant | - | $e^{(0.067\times BSA-0.0025\times Age-0.38\times Sex+1.7)}$ | 10.4 | [214] |
| Plasma weight | Non pregnant | - | $Blood weight*(1 - 0.91*HCT)$ |  |  |
| Plasma weight FC | Pregnant | <= 40 | $1.43E-02*GesAge + 1$ |  |  |
| Plasma weight FC | Postpartum | > 40 | $-4.16E-03*GesAge + 1.24$ |  |  |
| Plasma weight FC | Postpartum | > 57.7 | $1$ |  |  |
| Red blood cells weight | Non pregnant | - | $Blood weight - Plasma weight$ |  |  |
| Red blood cells weight FC | Pregnant | <= 40 | $2.47E-04*GesAge^{2} +2.49E-04*GesAge + 1$ |  |  |
| Red blood cells weight FC | Postpartum | > 40 | $2.07E-03*GesAge^{2} -1.95E-01*GesAge + 5.64$ |  |  |
| Red blood cells weight FC | Postpartum | > 46.5 | $1$ |  |  |
| Blood weight | Pregnant and postpartum |  | $Plasma weight*Plasma weight FC/(1-0.91*HCT)+RBC weight *RBC weight FC$ |  |  |
| Foetal weight | Pregnant | <= 40 | $((0.01*e^{\left( \frac{0.945}{0.0702} \right)*\left( 1 -\exp\left( -0.0702*GesAge \right) \right)}) / 1000) * 1.03$ | 27.51 | [132] |
| Foetal weight | Postpartum | > 40 | $0$ |  | [132] |
| Placental weight | Pregnant | <= 40 | $((-0.0122*GesAge^{3} +0.9149*GesAge^{2}-0.716*GesAge + 0) / 1000) * 1.048$ | 17.9 | [132] |
| Placental weight | Postpartum | > 40 | $0$ |  | [132] |
| Amniotic fluid weight | Pregnant | <= 40 | $0.00005*GesAge^{5}-0.0061*GesAge^{4}+0.2064*GesAge^{3} -1.2056*GesAge^{2} +1.9648*GesAge + 0) / 1000$ | 29.92 | [132] |
| Amniotic fluid weight | Postpartum | > 40 | $0$ |  | [132] |
| Foetal-placental weight | Pregnant and postpartum | All GA | $Foetal weight + Placental weight + Amniotic fluid weight$ |  | [132] |
| **Blood flow parameters** | | | | | |
| Cardiac output [L/h] | Non pregnant | - | $159 \times BSA-1.56 \times Age+114$ | 21.1 | [214] |
| Cardiac output FC | Pregnant and postpartum | <= 50.9 | $-4.77E-04*GesAge^{2}+ 2.43E-02*GesAge + 1$ |  |  |
| Cardiac output FC | Postpartum | > 50.9 | $1$ |  |  |
| Regional blood flows are expressed as % of cardiac output | | | | | |
| Adipose blood flow | Non pregnant | - | $(0.044+0.027\times Sex)\times Age+2.4\times Sex+3.9$ |  | [214] |
| Bone blood flow | Non pregnant | - | $5$ |  | [214] |
| Gonad blood flow | Non pregnant | - | $-0.03\times Sex+0.05$ |  | [214] |
| Heart blood flow | Non pregnant | - | $-0.72\times Body height-10\times Sex+134$ |  | [214] |
| Skin blood flow | Non pregnant | - | $5$ |  | [214] |
| Thymus blood flow | Non pregnant | - | $1.5$ |  | [214] |
| Liver blood flow | Non pregnant | - | $-0.108 \times Age+1.04 \times Sex+27.9$ |  | [214] |
| Gut blood flow | Non pregnant | - | $2\times Sex+14$ |  | [214] |
| Spleen blood flow | Non pregnant | - | $3$ |  | [214] |
| Pancreas blood flow | Non pregnant | - | $1$ |  | [214] |
| All blood flows above show no absolute change, therefore the % need to be adjusted accordingly to account for increase in cardiac output | | | | | |
| Blood flows FC | Pregnant | <= 40 | $-0.0099*GesAge + 1$ |  |  |
| Blood flows FC | Postpartum | > 40 | $0.0073*GesAge + 0.595$ |  |  |
| Blood flows FC | Postpartum | > 55 | $1$ |  |  |
| Remaining blood flows | | | | | |
| Brain blood flow | Non pregnant | - | $e^{-0.48\times BSA+0.04\times Sex+3.5}$ |  | [214] |
| Brain blood flow FC | Pregnant and postpartum | <= 82.4 | $(-1.24E-04*GesAge^{2} +1.13E-02*GesAge + 1) / Cardiac Output FC$ |  |  |
| Brain blood flow FC | Postpartum | > 82.4 | $1$ |  |  |
| Kidney blood flow | Non pregnant | - | $-8.7 \times BSA+0.29 \times Body height-0.081 \times Age-13$ |  | [214] |
| Kidney blood flow FC | Pregnant | <= 40.7 | $(-1.03E-03*GesAge^{2} +4.19E-02*GesAge + 1) / Cardiac Output FC$ |  |  |
| Kidney blood flow FC | Postpartum | > 40.7 | $1$ |  |  |
| Uterus blood flow | Non pregnant |  | $0.947$ |  | [132] |
| Uterus blood flow FC | Pregnant | <= 40 | $2.62E-03*GesAge^{2} +1.83E-01*GesAge + 1$ |  |  |
| Uterus blood flow FC | Postpartum | > 40 | $-0.66*GesAge + 36$ |  |  |
| Uterus blood flow FC | Postpartum | > 53 | $1$ |  |  |
| Muscle blood flow | Non pregnant | - | $-6.4 \times Sex+17.5$ |  | [214] |
| Muscle blood flow | Pregnant and postpartum | All GA | $(Muscle blood flow - Uterus blood flow) * Blood flows FC + Uterus blood flow * Uterus blood flow FC$ |  |  |
| Foetal-placental blood flow | Pregnant | > 2.23 and  < 40 | $-4.00E-04*GesAge^{3} +2.76E-02*GesAge^{2} -5.95E-02*GesAge + 0$ |  | [216] |
| **Other model parameters** | | | | | |
| GFR [mL/min] | Non pregnant | - | $e^{-0.0079\times Age+0.5\times BSA+4.2}$ | 14.7 | [214] |
| GFR FC | Pregnant and postpartum | <= 67.5 | $-2.33E-04*GesAge^{2}+1.57E-02*GesAge + 1$ |  |  |
| GFR FC | Postpartum | > 67.5 | $1$ |  |  |
| HCT | Non pregnant | - | $0.443 - 0.033*Sex$ | 14.4 | [214] |
| HCT FC | Pregnant and postpartum | <= 65 | $8.31E-05*GesAge^{2} - 5.37E-03*GesAge + 1$ |  |  |
| HCT FC | Postpartum | > 65 | $1$ |  |  |
| Albumin [g/L] | Non pregnant | - | $-0.0709*Age+47.7$ | 7.9 | [214] |
| Albumin FC | Pregnant | <= 40 | $1.75E-04*GesAge^{2}- 1.32E-02*GesAge + 1$ |  |  |
| Albumin FC | Postpartum | > 40 | $0.05*GesAge-1.23$ |  |  |
| Albumin FC | Postpartum | > 45 | $1$ |  |  |
| AAG [g/L] | Non pregnant | - | $0.798$ | 24.3 | [214] |
| AAG FC | Pregnant and postpartum | <= 63.7 | $2.07E-04*GesAge^{2} - 1.32E-02*GesAge + 1$ |  |  |
| AAG FC | Postpartum | > 63.7 | $1$ |  |  |
| **Liver and gastrointestinal enzymes abundance** | | | | | |
| CYP1A2 FC | Pregnant and postpartum | <= 51.8 | $9.54E-04*GesAge^{2}- 4.94E-02*GesAge + 1$ |  |  |
| CYP1A2 FC | Postpartum | > 51.8 | $1$ |  |  |
| CYP2B6 FC | Pregnant and postpartum | <= 50.8 | $-7.36E-04*GesAge^{2}+ 3.73E-02*GesAge + 1$ |  |  |
| CYP2B6 FC | Postpartum | > 50.8 | $1$ |  |  |
| CYP2C9 FC | Pregnant and postpartum | <= 42 | $-5.26E-03*GesAge^{2}+2.18E-01*GesAge + 1$ |  |  |
| CYP2C9 FC | Postpartum | > 42 | $1$ |  |  |
| CYP2C19 FC | Pregnant and postpartum | <= 43.5 | $-1.75E-03*GesAge^{2} +7.61E-02*GesAge + 1$ |  |  |
| CYP2C19 FC | Postpartum | > 43.5 | $1$ |  |  |
| CYP2D6 FC | Pregnant and postpartum | <= 51.4 | $-1.17E-03*GesAge^{2} +5.99E-02*GesAge + 1$ |  |  |
| CYP2D6 FC | Postpartum | > 51.4 | $1$ |  |  |
| CYP3A4 FC | Pregnant | <= 40 | $-1.11E-03*GesAge^{2} +6.23E-02*GesAge + 1$ |  |  |
| CYP3A4 FC | Postpartum | >40 | $-0.143*GesAge+7.44$ |  |  |
| CYP3A4 FC | Postpartum | > 45 | $1$ |  |  |
| CYP3A5 FC | Pregnant and postpartum | <= 53.4 | Assumed similar fold change as fort he CYP3A4 |  |  |
| CYP3A5 FC | Postpartum | > 53.4 | $1$ |  |  |
| UGT1A1 FC | Pregnant and postpartum | <= 46.5 | $-4.14E-04*GesAge^{2}+1.93E-02*GesAge + 1$ |  |  |
| UGT1A1 FC | Postpartum | > 46.5 | $1$ |  |  |
| UGT1A4 FC | Pregnant and postpartum | <= 52.1 | $-2.10E-03*GesAge^{2} +1.09E-01*GesAge + 1$ |  |  |
| UGT1A4 FC | Postpartum | > 52.1 | $1$ |  |  |
| Gastrointestinal tract enzymes |  |  | The same fold factor change is assumed to be the same also for enzymes expressed in the gastrointestinal tract |  |  |

*AAG* alpha-1 acid glycoprotein, *CV* coefficient of variance, *FC* fold change, *GesAge* gestational age, *GFR* glomerular filtration rate, *HCT* haematocrit, *RBC* red blood cells.

**Supplementary table 3**. Studies and demographic parameters (range) for the different antiretroviral drugs investigated in pregnant and postpartum women with HIV.

| **Drug** | **Dosing regimen** | **Second trimester** | | | | **Third trimester** | | | | **Postpartum** | | | |
| --- | --- | --- | --- | --- | --- | --- | --- | --- | --- | --- | --- | --- | --- |
|  |  | reference | n | Age | Gestational Age [weeks] | reference | n | Age | Gestational Age [weeks] | reference | n | Age | Gestational Age [weeks] |
|  |  |  |  | [years] |  |  |  | [years] |  |  |  | [years] |  |
| **Ritonavir** | 100mg QD S.S. | [150] | 14 | 18-33 | 24-28 | [150] | 14 | 18-33 | 34-38 | [150] | 14 | 18-33 | 46-52 |
| **Efavirenz** | 600mg QD S.S., PM and EM | [173] | 15 | 21-44 | 14-28 | [172, 173] | 65 | 19-44 | 28-39 | [172, 173] | 67 | 19-44 | 42-54 |
| **Efavirenz** | 600mg QD S.S., EM |  |  |  |  | [174] | 8 | 19-44 | 13-40 | [174] | 6 | 19-44 | 41-80 |
| **Rilpivirine** | 25mg QD S.S. | [201, 217] | 33 | 19-37 | 24-28 | [196, 201, 217] | 59 | 18-37 | 30-38 | [196, 201, 217] | 55 | 18-37 | 43-54 |
| **Doravirine** | 100mg QD S.S. | - | - | - | 13-27 | - | - | - | 28-40 | - | - | - | 46-52 |
| **Dolutegravir** | 50mg QD S.S. | [209] | 15 | 21-42 | 20-26 | [208, 209] | 71 | 21-42 | 27-38 | [208, 209] | 32 | 21-42 | 43-64 |
| **Bictegravir** | 50mg QD S.S. | [218, 219] | 31 | - | 13-27 | [218, 219] | 51 | - | 28-40 | [218, 219] | 74 | - | 46-52 |
| **Raltegravir** | 400mg BID S.S. | [207] | 16 | 19-43 | 20-26 | [206, 207] | 62 | 20-43 | 32-40 | [206, 207] | 56 | 20-43 | 43-51 |

*BID*, twice daily, *QD*, once daily, *S.S.*, steady state

**Supplementary figures**

**Supplementary figure 1.** Brain weight (a), heart weight (b), cardiac output (c), renal blood flow (d), glomerular filtration rate (e), and alpha-1 acid glycoprotein (f) versus gestational age. Black triangles represent the observed from the development dataset and red diamonds represent the data from the verification dataset. The grey circles represent the predicted datapoints calculated using the derived equations. Datapoints with multiple women are represented as mean ± standard deviation.


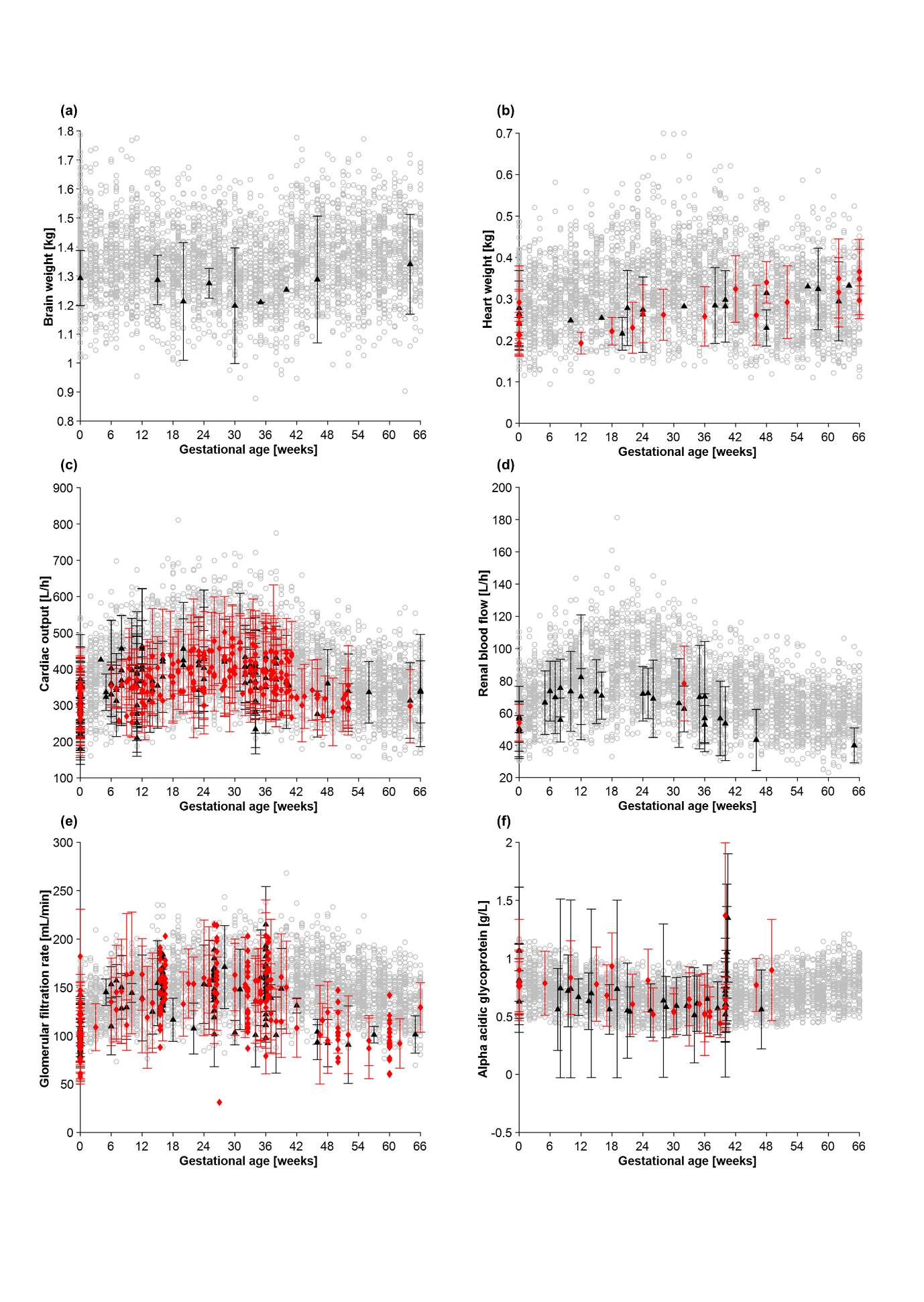


**Supplementary figure 2.** CYP1A2 (a), CYP2B6 (b), CYP2C9 (c), CYP2C19 (d), CYP2D6 (e), and UGT1A4 (f) abundance versus gestational age. Black triangles represent the observed from the development dataset and red diamonds represent the data from the verification dataset. The grey circles represent the predicted datapoints calculated using the derived equations. Datapoints with multiple women are represented as mean ± standard deviation.


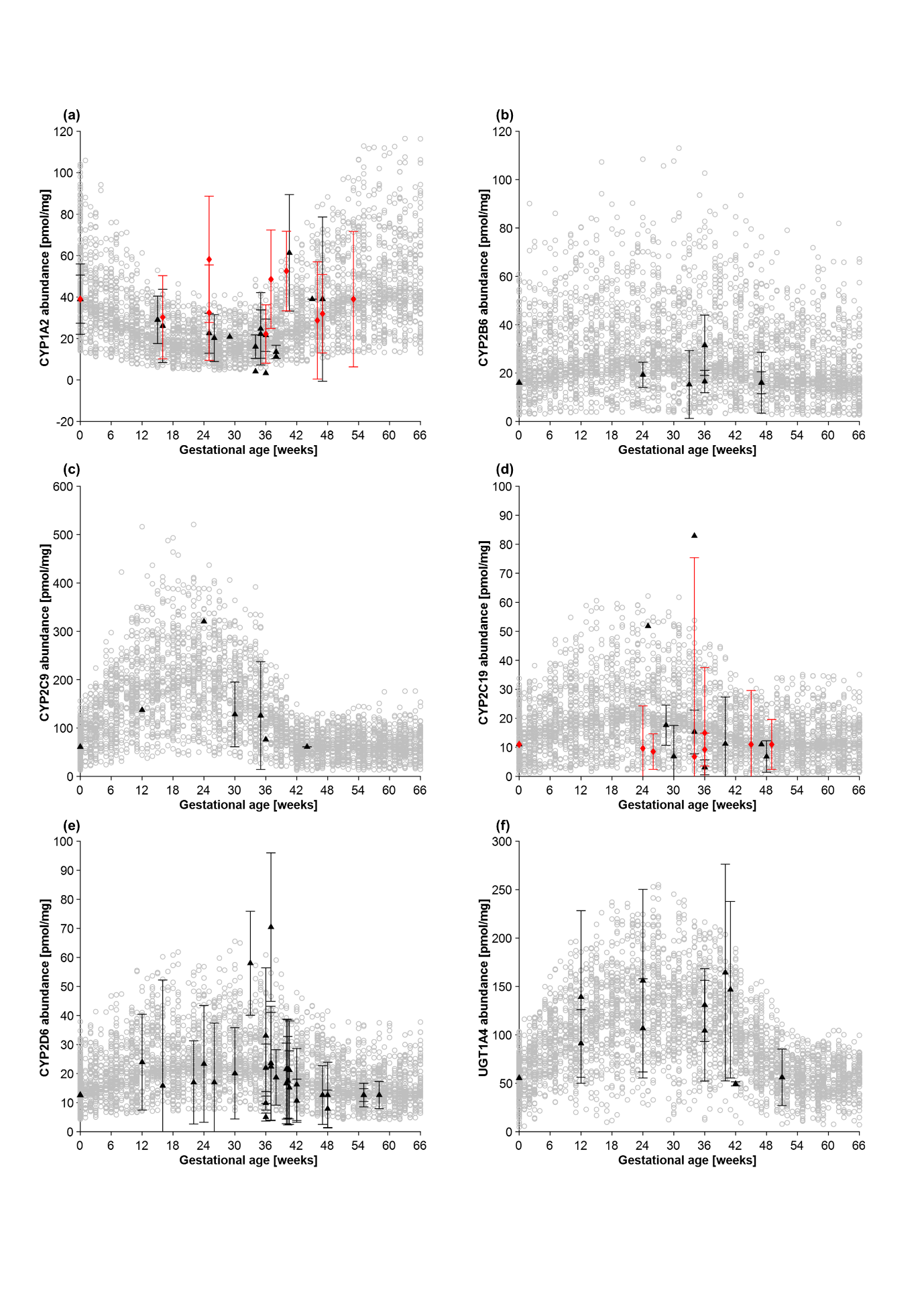


**Supplementary figure 3.** Concentration-time profile of ritonavir in pregnant women during the second trimester (a), third trimester (b) and postpartum women (c); and efavirenz in pregnant (d) and postpartum (e) women. Red circles represent the clinical observed data obtained from the literature. The solid bold line, solid lines, and shaded area represent the simulated mean of all virtual trials, the mean of each virtual trial, and the 90% normal range, respectively. The dashed lines represent the clinical efficacy threshold.


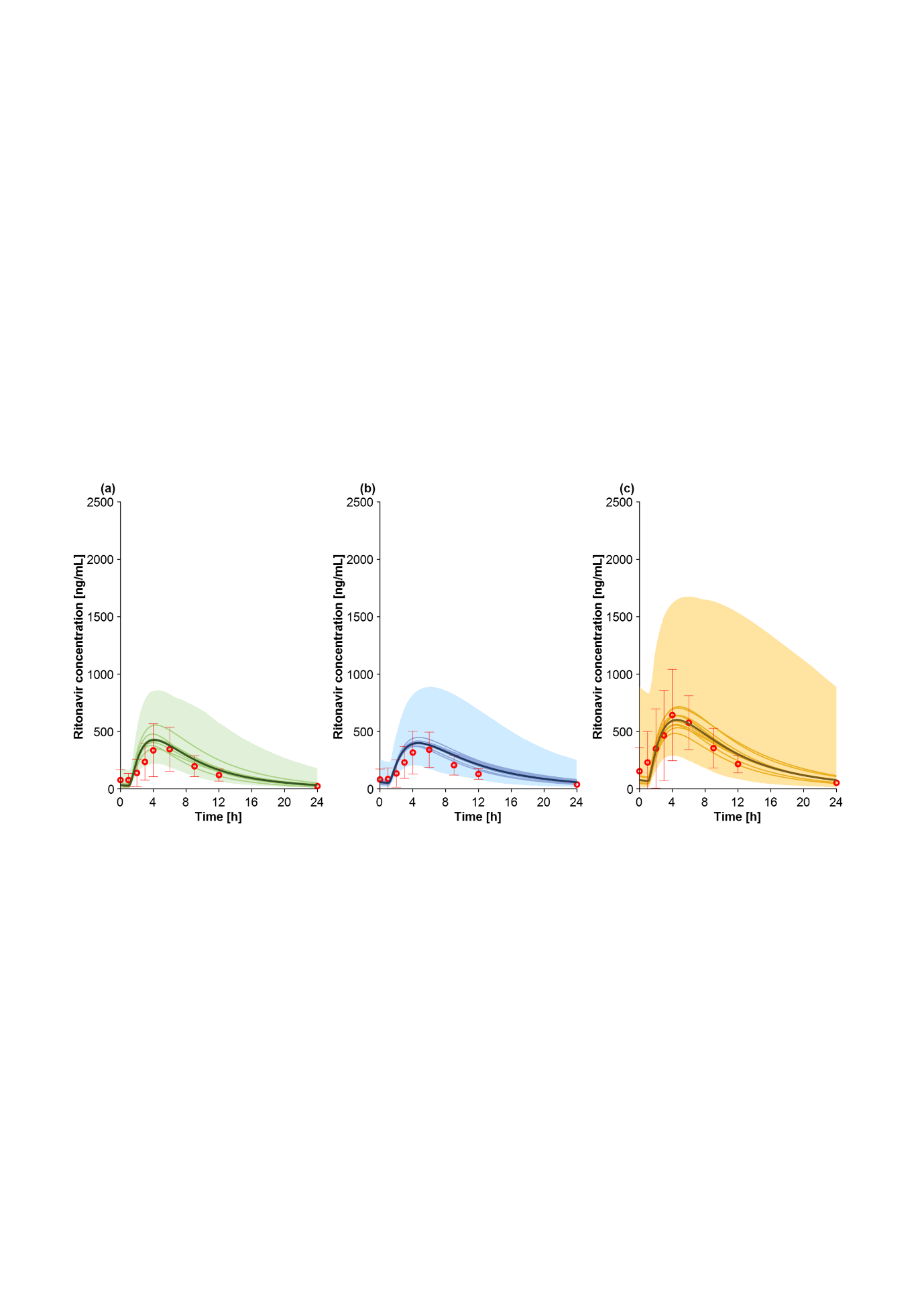


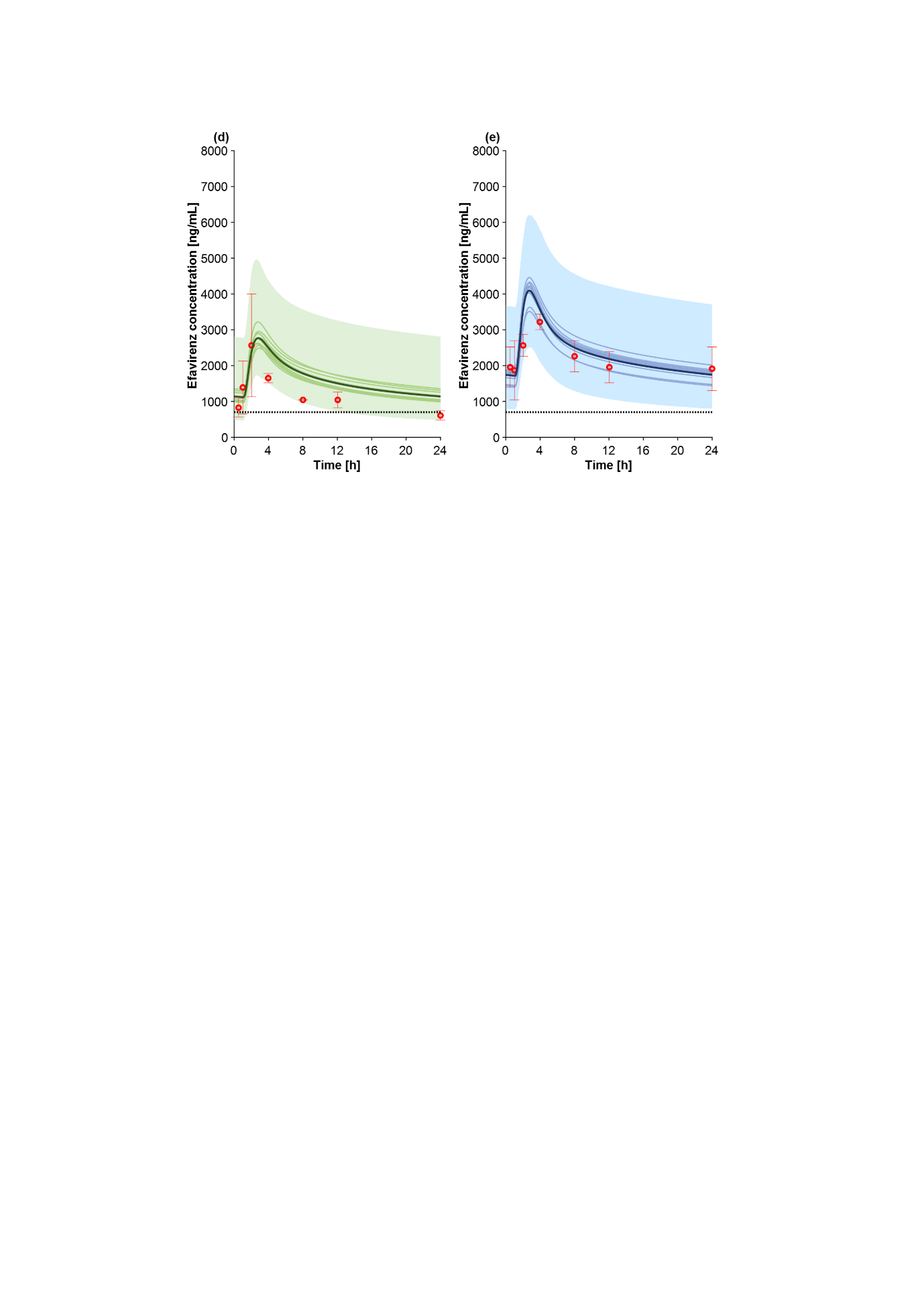


**References**

1. Emerson K, Jr., Poindexter EL, Kothari M. Changes in total body composition during normal and diabetic pregnancy. Relation to oxygen consumption. Obstet Gynecol **1975**; 45(5): 505-11.

2. Catalano PM, Roman-Drago NM, Amini SB, Sims EA. Longitudinal changes in body composition and energy balance in lean women with normal and abnormal glucose tolerance during pregnancy. Am J Obstet Gynecol **1998**; 179(1): 156-65.

3. Atkins AF, Watt JM, Milan P, Davies P, Crawford JS. A longitudinal study of cardiovascular dynamic changes throughout pregnancy. Eur J Obstet Gynecol Reprod Biol **1981**; 12(4): 215-24.

4. Spaanderman M, Ekhart T, van Eyck J, de Leeuw P, Peeters L. Preeclampsia and maladaptation to pregnancy: A role for atrial natriuretic peptide? Kidney Int **2001**; 60(4): 1397-406.

5. Kopp-Hoolihan LE, van Loan MD, Wong WW, King JC. Longitudinal assessment of energy balance in well-nourished, pregnant women. Am J Clin Nutr **1999**; 69(4): 697-704.

6. Petersen JW, Liu J, Chi YY, et al. Comparison of multiple non-invasive methods of measuring cardiac output during pregnancy reveals marked heterogeneity in the magnitude of cardiac output change between women. Physiol Rep **2017**; 5(8).

7. Chapman AB, Abraham WT, Zamudio S, et al. Temporal relationships between hormonal and hemodynamic changes in early human pregnancy. Kidney Int **1998**; 54(6): 2056-63.

8. de Groot LC, Boekholt HA, Spaaij CK, et al. Energy balances of healthy dutch women before and during pregnancy: Limited scope for metabolic adaptations in pregnancy. Am J Clin Nutr **1994**; 59(4): 827-32.

9. Spaanderman ME, Meertens M, van Bussel M, Ekhart TH, Peeters LL. Cardiac output increases independently of basal metabolic rate in early human pregnancy. Am J Physiol Heart Circ Physiol **2000**; 278(5): H1585-8.

10. Goldberg GR, Prentice AM, Coward WA, et al. Longitudinal assessment of energy expenditure in pregnancy by the doubly labeled water method. Am J Clin Nutr **1993**; 57(4): 494-505.

11. Robson SC, Hunter S, Boys RJ, Dunlop W. Serial study of factors influencing changes in cardiac output during human pregnancy. Am J Physiol **1989**; 256(4 Pt 2): H1060-5.

12. Edouard DA, Pannier BM, London GM, Cuche JL, Safar ME. Venous and arterial behavior during normal pregnancy. Am J Physiol **1998**; 274(5): H1605-12.

13. Lukaski HC, Siders WA, Nielsen EJ, Hall CB. Total body water in pregnancy: Assessment by using bioelectrical impedance. Am J Clin Nutr **1994**; 59(3): 578-85.

14. Rang S, de Pablo Lapiedra B, van Montfrans GA, Bouma BJ, Wesseling KH, Wolf H. Modelflow: A new method for noninvasive assessment of cardiac output in pregnant women. Am J Obstet Gynecol **2007**; 196(3): 235 e1-8.

15. Mabie WC, DiSessa TG, Crocker LG, Sibai BM, Arheart KL. A longitudinal study of cardiac output in normal human pregnancy. Am J Obstet Gynecol **1994**; 170(3): 849-56.

16. Caton D, Banner TE. Doppler estimates of cardiac output during pregnancy. Bull N Y Acad Med **1987**; 63(7): 727-31.

17. Forsum E, Sadurskis A, Wager J. Resting metabolic rate and body composition of healthy swedish women during pregnancy. Am J Clin Nutr **1988**; 47(6): 942-7.

18. Lof M, Olausson H, Bostrom K, Janerot-Sjoberg B, Sohlstrom A, Forsum E. Changes in basal metabolic rate during pregnancy in relation to changes in body weight and composition, cardiac output, insulin-like growth factor i, and thyroid hormones and in relation to fetal growth. Am J Clin Nutr **2005**; 81(3): 678-85.

19. Clapp JF, 3rd, Capeless E. Cardiovascular function before, during, and after the first and subsequent pregnancies. Am J Cardiol **1997**; 80(11): 1469-73.

20. Catalano PM, Wong WW, Drago NM, Amini SB. Estimating body composition in late gestation: A new hydration constant for body density and total body water. Am J Physiol **1995**; 268(1 Pt 1): E153-8.

21. Rookus MA, Rokebrand P, Burema J, Deurenberg P. The effect of pregnancy on the body mass index 9 months postpartum in 49 women. Int J Obes **1987**; 11(6): 609-18.

22. van Oppen AC, van der Tweel I, Alsbach GP, Heethaar RM, Bruinse HW. A longitudinal study of maternal hemodynamics during normal pregnancy. Obstet Gynecol **1996**; 88(1): 40-6.

23. Mahendru AA, Everett TR, Wilkinson IB, Lees CC, McEniery CM. A longitudinal study of maternal cardiovascular function from preconception to the postpartum period. J Hypertens **2014**; 32(4): 849-56.

24. Bamfo JE, Kametas NA, Nicolaides KH, Chambers JB. Maternal left ventricular diastolic and systolic long-axis function during normal pregnancy. Eur J Echocardiogr **2007**; 8(5): 360-8.

25. Butte NF, Hopkinson JM, Nicolson MA. Leptin in human reproduction: Serum leptin levels in pregnant and lactating women. J Clin Endocrinol Metab **1997**; 82(2): 585-9.

26. Thorsdottir I, Birgisdottir BE. Different weight gain in women of normal weight before pregnancy: Postpartum weight and birth weight. Obstet Gynecol **1998**; 92(3): 377-83.

27. Hronek M, Klemera P, Tosner J, Hrnciarikova D, Zadak Z. Anthropometric measured fat-free mass as essential determinant of resting energy expenditure for pregnant and non-pregnant women. Nutrition **2011**; 27(9): 885-90.

28. Gunderson EP, Abrams B, Selvin S. Does the pattern of postpartum weight change differ according to pregravid body size? Int J Obes Relat Metab Disord **2001**; 25(6): 853-62.

29. Smith DE, Lewis CE, Caveny JL, Perkins LL, Burke GL, Bild DE. Longitudinal changes in adiposity associated with pregnancy. The cardia study. Coronary artery risk development in young adults study. JAMA **1994**; 271(22): 1747-51.

30. Schauberger CW, Rooney BL, Brimer LM. Factors that influence weight loss in the puerperium. Obstet Gynecol **1992**; 79(3): 424-9.

31. Hatsu IE, McDougald DM, Anderson AK. Effect of infant feeding on maternal body composition. Int Breastfeed J **2008**; 3: 18.

32. Butte NF, Ellis KJ, Wong WW, Hopkinson JM, Smith EO. Composition of gestational weight gain impacts maternal fat retention and infant birth weight. Am J Obstet Gynecol **2003**; 189(5): 1423-32.

33. Langhoff-Roos J, Lindmark G, Gebre-Medhin M. Maternal fat stores and fat accretion during pregnancy in relation to infant birthweight. Br J Obstet Gynaecol **1987**; 94(12): 1170-7.

34. Durnin JV, McKillop FM, Grant S, Fitzgerald G. Energy requirements of pregnancy in scotland. Lancet **1987**; 2(8564): 897-900.

35. Ghezzi F, Franchi M, Balestreri D, et al. Bioelectrical impedance analysis during pregnancy and neonatal birth weight. Eur J Obstet Gynecol Reprod Biol **2001**; 98(2): 171-6.

36. Lederman SA, Paxton A, Heymsfield SB, Wang J, Thornton J, Pierson RN, Jr. Maternal body fat and water during pregnancy: Do they raise infant birth weight? Am J Obstet Gynecol **1999**; 180(1 Pt 1): 235-40.

37. Althuizen E, van der Wijden CL, van Mechelen W, Seidell JC, van Poppel MN. The effect of a counselling intervention on weight changes during and after pregnancy: A randomised trial. BJOG **2013**; 120(1): 92-9.

38. Lof M, Hilakivi-Clarke L, Sandin SS, de Assis S, Yu W, Weiderpass E. Dietary fat intake and gestational weight gain in relation to estradiol and progesterone plasma levels during pregnancy: A longitudinal study in swedish women. BMC Womens Health **2009**; 9: 10.

39. Linne Y, Barkeling B, Rossner S. Long-term weight development after pregnancy. Obes Rev **2002**; 3(2): 75-83.

40. Ohlin A, Rossner S. Maternal body weight development after pregnancy. Int J Obes **1990**; 14(2): 159-73.

41. Carmichael S, Abrams B, Selvin S. The pattern of maternal weight gain in women with good pregnancy outcomes. Am J Public Health **1997**; 87(12): 1984-8.

42. Greene GW, Smiciklas-Wright H, Scholl TO, Karp RJ. Postpartum weight change: How much of the weight gained in pregnancy will be lost after delivery? Obstet Gynecol **1988**; 71(5): 701-7.

43. Abrams B, Selvin S. Maternal weight gain pattern and birth weight. Obstet Gynecol **1995**; 86(2): 163-9.

44. Pipe NG, Smith T, Halliday D, Edmonds CJ, Williams C, Coltart TM. Changes in fat, fat-free mass and body water in human normal pregnancy. Br J Obstet Gynaecol **1979**; 86(12): 929-40.

45. Duvekot JJ, Cheriex EC, Pieters FA, Menheere PP, Peeters LH. Early pregnancy changes in hemodynamics and volume homeostasis are consecutive adjustments triggered by a primary fall in systemic vascular tone. Am J Obstet Gynecol **1993**; 169(6): 1382-92.

46. Lucini D, Strappazzon P, Dalla Vecchia L, Maggioni C, Pagani M. Cardiac autonomic adjustments to normal human pregnancy: Insight from spectral analysis of r-r interval and systolic arterial pressure variability. J Hypertens **1999**; 17(12 Pt 2): 1899-904.

47. Vered Z, Poler SM, Gibson P, Wlody D, Perez JE. Noninvasive detection of the morphologic and hemodynamic changes during normal pregnancy. Clin Cardiol **1991**; 14(4): 327-34.

48. Walters WA, MacGregor WG, Hills M. Cardiac output at rest during pregnancy and the puerperium. Clin Sci **1966**; 30(1): 1-11.

49. Hui AL, Back L, Ludwig S, et al. Effects of lifestyle intervention on dietary intake, physical activity level, and gestational weight gain in pregnant women with different pre-pregnancy body mass index in a randomized control trial. BMC Pregnancy Childbirth **2014**; 14: 331.

50. Mesa A, Jessurun C, Hernandez A, et al. Left ventricular diastolic function in normal human pregnancy. Circulation **1999**; 99(4): 511-7.

51. Hytten FE, Thomson AM, Taggart N. Total body water in normal pregnancy. J Obstet Gynaecol Br Commonw **1966**; 73(4): 553-61.

52. Easterling TR, Benedetti TJ, Schmucker BC, Millard SP. Maternal hemodynamics in normal and preeclamptic pregnancies: A longitudinal study. Obstet Gynecol **1990**; 76(6): 1061-9.

53. Villar J, Cogswell M, Kestler E, Castillo P, Menendez R, Repke JT. Effect of fat and fat-free mass deposition during pregnancy on birth weight. Am J Obstet Gynecol **1992**; 167(5): 1344-52.

54. Larciprete G, Valensise H, Vasapollo B, et al. Body composition during normal pregnancy: Reference ranges. Acta Diabetol **2003**; 40 Suppl 1: S225-32.

55. Britz SE, McDermott KC, Pierce CB, Blomquist JL, Handa VL. Changes in maternal weight 5-10 years after a first delivery. Womens Health (Lond) **2012**; 8(5): 513-9.

56. Robson SC, Hunter S, Moore M, Dunlop W. Haemodynamic changes during the puerperium: A doppler and m-mode echocardiographic study. Br J Obstet Gynaecol **1987**; 94(11): 1028-39.

57. Valensise H, Novelli GP, Vasapollo B, et al. Maternal cardiac systolic and diastolic function: Relationship with uteroplacental resistances. A doppler and echocardiographic longitudinal study. Ultrasound Obstet Gynecol **2000**; 15(6): 487-97.

58. Highman TJ, Friedman JE, Huston LP, Wong WW, Catalano PM. Longitudinal changes in maternal serum leptin concentrations, body composition, and resting metabolic rate in pregnancy. Am J Obstet Gynecol **1998**; 178(5): 1010-5.

59. Hopkinson JM, Butte NF, Ellis KJ, Wong WW, Puyau MR, Smith EO. Body fat estimation in late pregnancy and early postpartum: Comparison of two-, three-, and four-component models. Am J Clin Nutr **1997**; 65(2): 432-8.

60. Lof M, Forsum E. Hydration of fat-free mass in healthy women with special reference to the effect of pregnancy. Am J Clin Nutr **2004**; 80(4): 960-5.

61. van Raaij JM, Vermaat-Miedema SH, Schonk CM, Peek ME, Hautvast JG. Energy requirements of pregnancy in the Netherlands. Lancet **1987**; 2(8565): 953-5.

62. Van Loan MD, Kopp LE, King JC, Wong WW, Mayclin PL. Fluid changes during pregnancy: Use of bioimpedance spectroscopy. J Appl Physiol (1985) **1995**; 78(3): 1037-42.

63. Hutchinson DL, Plentl AA, Taylor HC, Jr. The total body water and the water turnover in pregnancy studied with deuterium oxide as isotopic tracer. J Clin Invest **1954**; 33(2): 235-41.

64. Ahmed SB, Bentley-Lewis R, Hollenberg NK, Graves SW, Seely EW. A comparison of prediction equations for estimating glomerular filtration rate in pregnancy. Hypertens Pregnancy **2009**; 28(3): 243-55.

65. Davison JM, Dunlop W, Ezimokhai M. 24-hour creatinine clearance during the third trimester of normal pregnancy. Br J Obstet Gynaecol **1980**; 87(2): 106-9.

66. Ezimokhai M, Davison JM, Philips PR, Dunlop W. Non-postural serial changes in renal function during the third trimester of normal human pregnancy. Br J Obstet Gynaecol **1981**; 88(5): 465-71.

67. Caton WL, Roby CC, et al. Plasma volume and extravascular fluid volume during pregnancy and the puerperium. Am J Obstet Gynecol **1949**; 57(3): 471-81.

68. Borghi C, Esposti DD, Immordino V, et al. Relationship of systemic hemodynamics, left ventricular structure and function, and plasma natriuretic peptide concentrations during pregnancy complicated by preeclampsia. Am J Obstet Gynecol **2000**; 183(1): 140-7.

69. Campbell DM, MacGillivray I. Comparison of maternal response in first and second pregnancies in relation to baby weight. J Obstet Gynaecol Br Commonw **1972**; 79(8): 684-93.

70. Haram K, Augensen K, Elsayed S. Serum protein pattern in normal pregnancy with special reference to acute-phase reactants. Br J Obstet Gynaecol **1983**; 90(2): 139-45.

71. Koetje PM, Spaan JJ, Kooman JP, Spaanderman ME, Peeters LL. Pregnancy reduces the accuracy of the estimated glomerular filtration rate based on cockroft-gault and mdrd formulas. Reprod Sci **2011**; 18(5): 456-62.

72. Mone SM, Sanders SP, Colan SD. Control mechanisms for physiological hypertrophy of pregnancy. Circulation **1996**; 94(4): 667-72.

73. Schannwell CM, Zimmermann T, Schneppenheim M, Plehn G, Marx R, Strauer BE. Left ventricular hypertrophy and diastolic dysfunction in healthy pregnant women. Cardiology **2002**; 97(2): 73-8.

74. Hutchins CJ. Plasma volume changes in pregnancy in indian and european primigravidae. Br J Obstet Gynaecol **1980**; 87(7): 586-9.

75. Lukaski HC, Hall CB, Siders WA. Assessment of change in hydration in women during pregnancy and postpartum with bioelectrical impedance vectors. Nutrition **2007**; 23(7-8): 543-50.

76. Munnell EW, Taylor HC. Liver blood flow in pregnancy-hepatic vein catheterization. J Clin Invest **1947**; 26(5): 952-6.

77. Pond SM, Kreek MJ, Tong TG, Raghunath J, Benowitz NL. Altered methadone pharmacokinetics in methadone-maintained pregnant women. J Pharmacol Exp Ther **1985**; 233(1): 1-6.

78. Robson SC, Mutch E, Boys RJ, Woodhouse KW. Apparent liver blood flow during pregnancy: A serial study using indocyanine green clearance. Br J Obstet Gynaecol **1990**; 97(8): 720-4.

79. Lund CJ, Donovan JC. Blood volume during pregnancy. Significance of plasma and red cell volumes. Am J Obstet Gynecol **1967**; 98(3): 394-403.

80. Sala C, Campise M, Ambroso G, Motta T, Zanchetti A, Morganti A. Atrial natriuretic peptide and hemodynamic changes during normal human pregnancy. Hypertension **1995**; 25(4 Pt 1): 631-6.

81. Cohen ME, Thomson KJ. Studies on the circulation in pregnancy. I. The velocity of blood flow and related aspects of the circulation in normal pregnant women. J Clin Invest **1936**; 15(6): 607-25.

82. Weir RJ, Paintin DB, Brown JJ, et al. A serial study in pregnancy of the plasma concentrations of renin, corticosteroids, electrolytes and proteins and of haematocrit and plasma volume. J Obstet Gynaecol Br Commonw **1971**; 78(7): 590-602.

83. Frederiksen MC, Ruo TI, Chow MJ, Atkinson AJ, Jr. Theophylline pharmacokinetics in pregnancy. Clin Pharmacol Ther **1986**; 40(3): 321-8.

84. Roberts M, Lindheimer MD, Davison JM. Altered glomerular permselectivity to neutral dextrans and heteroporous membrane modeling in human pregnancy. Am J Physiol **1996**; 270(2 Pt 2): F338-43.

85. Dunlop W. Serial changes in renal haemodynamics during normal human pregnancy. Br J Obstet Gynaecol **1981**; 88(1): 1-9.

86. Salas SP, Marshall G, Gutierrez BL, Rosso P. Time course of maternal plasma volume and hormonal changes in women with preeclampsia or fetal growth restriction. Hypertension **2006**; 47(2): 203-8.

87. Hebert MF, Easterling TR, Kirby B, et al. Effects of pregnancy on cyp3a and p-glycoprotein activities as measured by disposition of midazolam and digoxin: A university of washington specialized center of research study. Clin Pharmacol Ther **2008**; 84(2): 248-53.

88. Bruinse HW, van den Berg H, Haspels AA. Smoking and its effect on maternal plasma volume during and after normal pregnancy. Eur J Obstet Gynecol Reprod Biol **1985**; 20(4): 215-9.

89. Kopp-Hoolihan LE, van Loan MD, Wong WW, King JC. Fat mass deposition during pregnancy using a four-component model. J Appl Physiol (1985) **1999**; 87(1): 196-202.

90. Lederman SA, Paxton A, Heymsfield SB, Wang J, Thornton J, Pierson RN, Jr. Body fat and water changes during pregnancy in women with different body weight and weight gain. Obstet Gynecol **1997**; 90(4 Pt 1): 483-8.

91. Seitchik J. Total body water and total body density of pregnant women. Obstet Gynecol **1967**; 29(2): 155-66.

92. Burwell CS, Strayhorn WD, Flickinger D, Corlette MB, Bowerman EP, Kennedy JA. Circulation during pregnancy. Archives of Internal Medicine **1938**; 62(6): 979-1003.

93. Oatridge A, Holdcroft A, Saeed N, et al. Change in brain size during and after pregnancy: Study in healthy women and women with preeclampsia. AJNR Am J Neuroradiol **2002**; 23(1): 19-26.

94. Kametas NA, McAuliffe F, Hancock J, Chambers J, Nicolaides KH. Maternal left ventricular mass and diastolic function during pregnancy. Ultrasound Obstet Gynecol **2001**; 18(5): 460-6.

95. Poppas A, Shroff SG, Korcarz CE, et al. Serial assessment of the cardiovascular system in normal pregnancy. Role of arterial compliance and pulsatile arterial load. Circulation **1997**; 95(10): 2407-15.

96. Christensen T, Klebe JG, Bertelsen V, Hansen HE. Changes in renal volume during normal pregnancy. Acta Obstet Gynecol Scand **1989**; 68(6): 541-3.

97. Bailey RR, Rolleston GL. Kidney length and ureteric dilatation in the puerperium. J Obstet Gynaecol Br Commonw **1971**; 78(1): 55-61.

98. Low JA, Johnston EE, McBride RL. Blood volume adjustments in the normal obstetric patient with particular reference to the third trimester of pregnancy. Am J Obstet Gynecol **1965**; 91: 356-63.

99. Bucht H. Studies on renal function in man; with special reference to glomerular filtration and renal plasma flow in pregnancy. Scand J Clin Lab Invest **1951**; 3 Suppl. 3: 1-64.

100. Pirani BB, MacGillivray I. Smoking during pregnancy. Its effect on maternal metabolism and fetoplacental function. Obstet Gynecol **1978**; 52(3): 257-63.

101. Tuttle S, Aggett PJ, Campbell D, MacGillivray I. Zinc and copper nutrition in human pregnancy: A longitudinal study in normal primigravidae and in primigravidae at risk of delivering a growth retarded baby. Am J Clin Nutr **1985**; 41(5): 1032-41.

102. Verel D, Bury JD, Hope A. Blood volume changes in pregnancy and the puerperium. Clin Sci **1956**; 15(1): 1-7.

103. Gibson HM. Plasma volume and glomerular filtration rate in pregnancy and their relation to differences in fetal growth. J Obstet Gynaecol Br Commonw **1973**; 80(12): 1067-74.

104. Whittaker PG, Lind T. The intravascular mass of albumin during human pregnancy: A serial study in normal and diabetic women. Br J Obstet Gynaecol **1993**; 100(6): 587-92.

105. Whittaker PG, Macphail S, Lind T. Serial hematologic changes and pregnancy outcome. Obstet Gynecol **1996**; 88(1): 33-9.

106. Taylor DJ, Lind T. Red cell mass during and after normal pregnancy. Br J Obstet Gynaecol **1979**; 86(5): 364-70.

107. Pirani BB, Campbell DM, MacGillivray I. Plasma volume in normal first pregnancy. J Obstet Gynaecol Br Commonw **1973**; 80(10): 884-7.

108. Hytten FE, Paintin DB. Increase in plasma volume during normal pregnancy. J Obstet Gynaecol Br Emp **1963**; 70: 402-7.

109. Pritchard JA. Changes in the blood volume during pregnancy and delivery. Anesthesiology **1965**; 26: 393-9.

110. Adams JQ. Cardiovascular physiology in normal pregnancy: Studies with the dye dilution technique. Am J Obstet Gynecol **1954**; 67(4): 741-59.

111. Rovinsky JJ, Jaffin H. Cardiovascular hemodynamics in pregnancy. I. Blood and plasma volumes in multiple pregnancy. Am J Obstet Gynecol **1965**; 93: 1-15.

112. Bernstein IM, Ziegler W, Badger GJ. Plasma volume expansion in early pregnancy. Obstet Gynecol **2001**; 97(5 Pt 1): 669-72.

113. Ueland K. Maternal cardiovascular dynamics. Vii. Intrapartum blood volume changes. Am J Obstet Gynecol **1976**; 126(6): 671-7.

114. MacLennan FM, MacDonald AF, Campbell DM. Lung water during the puerperium. Anaesthesia **1987**; 42(2): 141-7.

115. Clark SL, Cotton DB, Pivarnik JM, et al. Position change and central hemodynamic profile during normal third-trimester pregnancy and post partum. Am J Obstet Gynecol **1991**; 164(3): 883-7.

116. Bader RA, Bader ME, Rose DF, Braunwald E. Hemodynamics at rest and during exercise in normal pregnancy as studies by cardiac catheterization. J Clin Invest **1955**; 34(10): 1524-36.

117. Vinayagam D, Thilaganathan B, Stirrup O, Mantovani E, Khalil A. Maternal hemodynamics in normal pregnancy: Reference ranges and role of maternal characteristics. Ultrasound Obstet Gynecol **2018**; 51(5): 665-71.

118. Hale SA, Schonberg A, Badger GJ, Bernstein IM. Relationship between prepregnancy and early pregnancy uterine blood flow and resistance index. Reprod Sci **2009**; 16(11): 1091-6.

119. Hennessy TG, MacDonald D, Hennessy MS, et al. Serial changes in cardiac output during normal pregnancy: A doppler ultrasound study. Eur J Obstet Gynecol Reprod Biol **1996**; 70(2): 117-22.

120. Del Bene R, Barletta G, Mello G, et al. Cardiovascular function in pregnancy: Effects of posture. BJOG **2001**; 108(4): 344-52.

121. Kametas NA, McAuliffe F, Cook B, Nicolaides KH, Chambers J. Maternal left ventricular transverse and long-axis systolic function during pregnancy. Ultrasound Obstet Gynecol **2001**; 18(5): 467-74.

122. Rubler S, Damani PM, Pinto ER. Cardiac size and performance during pregnancy estimated with echocardiography. Am J Cardiol **1977**; 40(4): 534-40.

123. Capeless EL, Clapp JF. Cardiovascular changes in early phase of pregnancy. Am J Obstet Gynecol **1989**; 161(6 Pt 1): 1449-53.

124. Capeless EL, Clapp JF. When do cardiovascular parameters return to their preconception values? Am J Obstet Gynecol **1991**; 165(4 Pt 1): 883-6.

125. Katz R, Karliner JS, Resnik R. Effects of a natural volume overload state (pregnancy) on left ventricular performance in normal human subjects. Circulation **1978**; 58(3 Pt 1): 434-41.

126. Hamilton HF. Blood viscosity in pregnancy. J Obstet Gynaecol Br Emp **1950**; 57(4): 530-8.

127. Nevo O, Soustiel JF, Thaler I. Maternal cerebral blood flow during normal pregnancy: A cross-sectional study. Am J Obstet Gynecol **2010**; 203(5): 475 e1-6.

128. Zeeman GG, Hatab M, Twickler DM. Maternal cerebral blood flow changes in pregnancy. Am J Obstet Gynecol **2003**; 189(4): 968-72.

129. Irons DW, Baylis PH, Davison JM. Effect of atrial natriuretic peptide on renal hemodynamics and sodium excretion during human pregnancy. Am J Physiol **1996**; 271(1 Pt 2): F239-42.

130. Sims EA, Krantz KE. Serial studies of renal function during pregnancy and the puerperium in normal women. J Clin Invest **1958**; 37(12): 1764-74.

131. Chesley LC, Connell EJ, Chesley ER, Katz JD, Glissen CS. The diodrast clearance and renal blood flow in toxemias of pregnancy. J Clin Invest **1940**; 19(1): 219-24.

132. Abduljalil K, Furness P, Johnson TN, Rostami-Hodjegan A, Soltani H. Anatomical, physiological and metabolic changes with gestational age during normal pregnancy: A database for parameters required in physiologically based pharmacokinetic modelling. Clin Pharmacokinet **2012**; 51(6): 365-96.

133. Semple PF, Carswell W, Boyle JA. Serial studies of the renal clearance of urate and inulin during pregnancy and after the puerperium in normal women. Clin Sci Mol Med **1974**; 47(6): 559-65.

134. Davison JM, Dunlop W. Renal hemodynamics and tubular function normal human pregnancy. Kidney Int **1980**; 18(2): 152-61.

135. Moran P, Baylis PH, Lindheimer MD, Davison JM. Glomerular ultrafiltration in normal and preeclamptic pregnancy. J Am Soc Nephrol **2003**; 14(3): 648-52.

136. Sturgiss SN, Wilkinson R, Davison JM. Renal reserve during human pregnancy. Am J Physiol **1996**; 271(1 Pt 2): F16-20.

137. Dunlop W. Investigations into the influence of posture on renal plasma flow and glomerular filtration rate during late pregnancy. Br J Obstet Gynaecol **1976**; 83(1): 17-23.

138. Milne JE, Lindheimer MD, Davison JM. Glomerular heteroporous membrane modeling in third trimester and postpartum before and during amino acid infusion. Am J Physiol Renal Physiol **2002**; 282(1): F170-5.

139. De Alvarez RR. Renal glomerulotubular mechanisms during normal pregnancy. I. Glomerular filtration rate, renal plasma flow, and creatinine clearance. Am J Obstet Gynecol **1958**; 75(5): 931-44.

140. Saxena AR, Ananth Karumanchi S, Fan SL, et al. Correlation of cystatin-c with glomerular filtration rate by inulin clearance in pregnancy. Hypertens Pregnancy **2012**; 31(1): 22-30.

141. Ronco C, Brendolan A, Bragantini L, et al. Renal functional reserve in pregnancy. Nephrol Dial Transplant **1988**; 3(2): 157-61.

142. Assali NS, Dignam WJ, Dasgupta K. Renal function in human pregnancy. Ii. Effects of venous pooling on renal hemodynamics and water, electrolyte, and aldosterone excretion during gestation. J Lab Clin Med **1959**; 54: 394-408.

143. Colbers A, Greupink R, Litjens C, Burger D, Russel FG. Physiologically based modelling of darunavir/ritonavir pharmacokinetics during pregnancy. Clin Pharmacokinet **2016**; 55(3): 381-96.

144. Anim-Nyame N, Sooranna SR, Johnson MR, Gamble J, Steer PJ. Resting peripheral blood flow in normal pregnancy and in pre-eclampsia. Clin Sci (Lond) **2000**; 99(6): 505-10.

145. Chanarin I, Rothman D, Berry V. Iron deficiency and its relation to folic-acid status in pregnancy: Results of a clinical trial. Br Med J **1965**; 1(5433): 480-5.

146. Wu PY, Udani V, Chan L, Miller FC, Henneman CE. Colloid osmotic pressure: Variations in normal pregnancy. J Perinat Med **1983**; 11(4): 193-9.

147. Krauer B, Dayer P, Anner R. Changes in serum albumin and alpha 1-acid glycoprotein concentrations during pregnancy: An analysis of fetal-maternal pairs. Br J Obstet Gynaecol **1984**; 91(9): 875-81.

148. Larijani GE, Norris MC, Ala-Kokko TI, Leighton BA, DeSimone C. Serum concentration of alpha 1-acid glycoprotein and albumin following cesarean section and vaginal delivery. DICP **1990**; 24(3): 328-9.

149. Tsen LC, Tarshis J, Denson DD, Osathanondh R, Datta S, Bader AM. Measurements of maternal protein binding of bupivacaine throughout pregnancy. Anesth Analg **1999**; 89(4): 965-8.

150. Crauwels HM, Kakuda TN, Ryan B, et al. Pharmacokinetics of once-daily darunavir/ritonavir in HIV-1-infected pregnant women. HIV Med **2016**; 17(9): 643-52.

151. Paaby P. Changes in serum proteins during pregnancy. J Obstet Gynaecol Br Emp **1960**; 67: 43-55.

152. Von Studnitz W. Studies on serum proteins in pregnancy. Scand J Clin Lab Invest **1955**; 7(4): 324-8.

153. Beetham R, Dawnay A, Menabawy M, Silver A. Urinary excretion of albumin and retinol-binding protein during normal pregnancy. J Clin Pathol **1988**; 41(10): 1089-92.

154. Horne CH, Howie PW, Goudie RB. Serum-alpha2-macroglobulin, transferrin, albumin, and igg levels in preeclampsia. J Clin Pathol **1970**; 23(6): 514-6.

155. Macgillivray I, Tovey JE. A study of the serum protein changes in pregnancy and toxaemia, using paper strip electrophoresis. J Obstet Gynaecol Br Emp **1957**; 64(3): 361-4.

156. Dean M, Stock B, Patterson RJ, Levy G. Serum protein binding of drugs during and after pregnancy in humans. Clin Pharmacol Ther **1980**; 28(2): 253-61.

157. Connelly TJ, Ruo TI, Frederiksen MC, Atkinson AJ, Jr. Characterization of theophylline binding to serum proteins in pregnant and nonpregnant women. Clin Pharmacol Ther **1990**; 47(1): 68-72.

158. Mendenhall HW. Serum protein concentrations in pregnancy. I. Concentrations in maternal serum. Am J Obstet Gynecol **1970**; 106(3): 388-99.

159. Pitkin RM, Reynolds WA, Williams GA, Hargis GK. Calcium metabolism in normal pregnancy: A longitudinal study. Am J Obstet Gynecol **1979**; 133(7): 781-90.

160. Succari M, Foglietti MJ, Percheron F. Microheterogeneity of alpha 1-acid glycoprotein: Variation during the menstrual cycle in healthy women, and profile in women receiving estrogen-progestogen treatment. Clin Chim Acta **1990**; 187(3): 235-41.

161. Havenaar EC, Axford JS, Brinkman-van der Linden EC, et al. Severe rheumatoid arthritis prohibits the pregnancy-induced decrease in alpha3-fucosylation of alpha1-acid glycoprotein. Glycoconj J **1998**; 15(7): 723-9.

162. Kovar IZ, Riches PG. C3 and c4 complement components and acute phase proteins in late pregnancy and parturition. J Clin Pathol **1988**; 41(6): 650-2.

163. Chu CY, Singla VP, Wang HP, Sweet B, Lai LT. Plasma alpha 1-acid glycoprotein levels in pregnancy. Clin Chim Acta **1981**; 112(2): 235-40.

164. Bologa M, Tang B, Klein J, Tesoro A, Koren G. Pregnancy-induced changes in drug metabolism in epileptic women. J Pharmacol Exp Ther **1991**; 257(2): 735-40.

165. Brazier JL, Ritter J, Berland M, Khenfer D, Faucon G. Pharmacokinetics of caffeine during and after pregnancy. Dev Pharmacol Ther **1983**; 6(5): 315-22.

166. Tsutsumi K, Kotegawa T, Matsuki S, et al. The effect of pregnancy on cytochrome p4501a2, xanthine oxidase, and n-acetyltransferase activities in humans. Clin Pharmacol Ther **2001**; 70(2): 121-5.

167. Knutti R, Rothweiler H, Schlatter C. Effect of pregnancy on the pharmacokinetics of caffeine. Eur J Clin Pharmacol **1981**; 21(2): 121-6.

168. Scott NR, Chakraborty J, Marks V. Urinary metabolites of caffeine in pregnant women. Br J Clin Pharmacol **1986**; 22(4): 475-8.

169. Gardner MJ, Schatz M, Cousins L, Zeiger R, Middleton E, Jusko WJ. Longitudinal effects of pregnancy on the pharmacokinetics of theophylline. Eur J Clin Pharmacol **1987**; 32(3): 289-95.

170. Romero R, Kadar N, Gonzales Govea F, Hobbins JC. Pharmacokinetics of intravenous theophylline in pregnant patients at term. Am J Perinatol **1983**; 1(1): 31-5.

171. Tracy TS, Venkataramanan R, Glover DD, Caritis SN, National Institute for Child H, Human Development Network of Maternal-Fetal-Medicine U. Temporal changes in drug metabolism (cyp1a2, cyp2d6 and cyp3a activity) during pregnancy. Am J Obstet Gynecol **2005**; 192(2): 633-9.

172. Cressey TR, Stek A, Capparelli E, et al. Efavirenz pharmacokinetics during the third trimester of pregnancy and postpartum. J Acquir Immune Defic Syndr **2012**; 59(3): 245-52.

173. Kreitchmann R, Schalkwijk S, Best B, et al. Efavirenz pharmacokinetics during pregnancy and infant washout. Antivir Ther **2019**; 24(2): 95-103.

174. Olagunju A, Bolaji O, Amara A, et al. Pharmacogenetics of pregnancy-induced changes in efavirenz pharmacokinetics. Clin Pharmacol Ther **2015**; 97(3): 298-306.

175. Yerby MS, Friel PN, McCormick K, et al. Pharmacokinetics of anticonvulsants in pregnancy: Alterations in plasma protein binding. Epilepsy Res **1990**; 5(3): 223-8.

176. Hebert MF, Ma X, Naraharisetti SB, et al. Are we optimizing gestational diabetes treatment with glyburide? The pharmacologic basis for better clinical practice. Clin Pharmacol Ther **2009**; 85(6): 607-14.

177. Wangboonskul J, White NJ, Nosten F, ter Kuile F, Moody RR, Taylor RB. Single dose pharmacokinetics of proguanil and its metabolites in pregnancy. Eur J Clin Pharmacol **1993**; 44(3): 247-51.

178. McGready R, Stepniewska K, Edstein MD, et al. The pharmacokinetics of atovaquone and proguanil in pregnant women with acute falciparum malaria. Eur J Clin Pharmacol **2003**; 59(7): 545-52.

179. Hirt D, Treluyer JM, Jullien V, et al. Pregnancy-related effects on nelfinavir-m8 pharmacokinetics: A population study with 133 women. Antimicrob Agents Chemother **2006**; 50(6): 2079-86.

180. Villani P, Floridia M, Pirillo MF, et al. Pharmacokinetics of nelfinavir in HIV-1-infected pregnant and nonpregnant women. Br J Clin Pharmacol **2006**; 62(3): 309-15.

181. Read JS, Best BM, Stek AM, et al. Pharmacokinetics of new 625 mg nelfinavir formulation during pregnancy and postpartum. HIV Med **2008**; 9(10): 875-82.

182. Mulligan N, Schalkwijk S, Best BM, et al. Etravirine pharmacokinetics in HIV-infected pregnant women. Front Pharmacol **2016**; 7: 239.

183. Ramgopal M, Osiyemi O, Zorrilla C, et al. Pharmacokinetics of total and unbound etravirine in HIV-1-infected pregnant women. J Acquir Immune Defic Syndr **2016**; 73(3): 268-74.

184. Izurieta P KT, Clark A, Feys C, Witek J. Safety and pharmacokinetics of etravirine in pregnant HIV-infected women. 12th European AIDS Conference. Cologne, Germany, **2009**.

185. Hogstedt S, Lindberg B, Peng DR, Regardh CG, Rane A. Pregnancy-induced increase in metoprolol metabolism. Clin Pharmacol Ther **1985**; 37(6): 688-92.

186. Hogstedt S, Lindberg B, Rane A. Increased oral clearance of metoprolol in pregnancy. Eur J Clin Pharmacol **1983**; 24(2): 217-20.

187. Ryu RJ, Eyal S, Easterling TR, et al. Pharmacokinetics of metoprolol during pregnancy and lactation. J Clin Pharmacol **2016**; 56(5): 581-9.

188. Wadelius M, Darj E, Frenne G, Rane A. Induction of cyp2d6 in pregnancy. Clin Pharmacol Ther **1997**; 62(4): 400-7.

189. Heikkinen T, Ekblad U, Palo P, Laine K. Pharmacokinetics of fluoxetine and norfluoxetine in pregnancy and lactation. Clin Pharmacol Ther **2003**; 73(4): 330-7.

190. Heikkinen T, Ekblad U, Kero P, Ekblad S, Laine K. Citalopram in pregnancy and lactation. Clin Pharmacol Ther **2002**; 72(2): 184-91.

191. Cressey TR, Best BM, Achalapong J, et al. Reduced indinavir exposure during pregnancy. Br J Clin Pharmacol **2013**; 76(3): 475-83.

192. Acosta EP, Bardeguez A, Zorrilla CD, et al. Pharmacokinetics of saquinavir plus low-dose ritonavir in human immunodeficiency virus-infected pregnant women. Antimicrob Agents Chemother **2004**; 48(2): 430-6.

193. Ripamonti D, Cattaneo D, Maggiolo F, et al. Atazanavir plus low-dose ritonavir in pregnancy: Pharmacokinetics and placental transfer. AIDS **2007**; 21(18): 2409-15.

194. Conradie F, Zorrilla C, Josipovic D, et al. Safety and exposure of once-daily ritonavir-boosted atazanavir in HIV-infected pregnant women. HIV Med **2011**; 12(9): 570-9.

195. Colbers A, Molto J, Ivanovic J, et al. Pharmacokinetics of total and unbound darunavir in HIV-1-infected pregnant women. J Antimicrob Chemother **2015**; 70(2): 534-42.

196. Schalkwijk S, Colbers A, Konopnicki D, et al. Lowered rilpivirine exposure during the third trimester of pregnancy in human immunodeficiency virus type 1-infected women. Clin Infect Dis **2017**; 65(8): 1335-41.

197. Colbers A, Gingelmaier A, van der Ende M, Rijnders B, Burger D. Pharmacokinetics, safety and transplacental passage of rilpivirine in pregnancy: Two cases. AIDS **2014**; 28(2): 288-90.

198. Crauwels HM, Osiyemi O, Zorrilla C, Bicer C, Brown K. Reduced exposure to darunavir and cobicistat in HIV-1-infected pregnant women receiving a darunavir/cobicistat-based regimen. HIV Med **2019**; 20(5): 337-43.

199. Momper JD, Best BM, Wang J, et al. Elvitegravir/cobicistat pharmacokinetics in pregnant and postpartum women with HIV. AIDS **2018**; 32(17): 2507-16.

200. Mirochnick M, Best BM, Stek AM, et al. Atazanavir pharmacokinetics with and without tenofovir during pregnancy. J Acquir Immune Defic Syndr **2011**; 56(5): 412-9.

201. Tran AH, Best BM, Stek A, et al. Pharmacokinetics of rilpivirine in HIV-infected pregnant women. J Acquir Immune Defic Syndr **2016**; 72(3): 289-96.

202. Colbers A, Hawkins D, Hidalgo-Tenorio C, et al. Atazanavir exposure is effective during pregnancy regardless of tenofovir use. Antivir Ther **2015**; 20(1): 57-64.

203. Kreitchmann R, Best BM, Wang J, et al. Pharmacokinetics of an increased atazanavir dose with and without tenofovir during the third trimester of pregnancy. J Acquir Immune Defic Syndr **2013**; 63(1): 59-66.

204. Zorrilla CD, Wright R, Osiyemi OO, et al. Total and unbound darunavir pharmacokinetics in pregnant women infected with HIV-1: Results of a study of darunavir/ritonavir 600/100 mg administered twice daily. HIV Med **2014**; 15(1): 50-6.

205. Colbers A, Best B, Schalkwijk S, et al. Maraviroc pharmacokinetics in HIV-1-infected pregnant women. Clin Infect Dis **2015**; 61(10): 1582-9.

206. Blonk MI, Colbers AP, Hidalgo-Tenorio C, et al. Raltegravir in HIV-1-infected pregnant women: Pharmacokinetics, safety, and efficacy. Clin Infect Dis **2015**; 61(5): 809-16.

207. Watts DH, Stek A, Best BM, et al. Raltegravir pharmacokinetics during pregnancy. J Acquir Immune Defic Syndr **2014**; 67(4): 375-81.

208. Bollen P, Freriksen J, Konopnicki D, et al. The effect of pregnancy on the pharmacokinetics of total and unbound dolutegravir and its main metabolite in women living with human immunodeficiency virus. Clin Infect Dis **2021**; 72(1): 121-7.

209. Mulligan N, Best BM, Wang J, et al. Dolutegravir pharmacokinetics in pregnant and postpartum women living with HIV. AIDS **2018**; 32(6): 729-37.

210. Whitehead EM, Smith M, Dean Y, O'Sullivan G. An evaluation of gastric emptying times in pregnancy and the puerperium. Anaesthesia **1993**; 48(1): 53-7.

211. Orell C, Kintu K, Coombs JA, et al. Dolphin-1: Randomised controlled trial of dolutegravir(dtg)-versus efavirenz(efv)-based therapy in mothers initiating antiretroviral treatment in late pregnancy. 22nd International AIDS Conference. Amsterdam, Netherlands **2018**.

212. Fotopoulou C, Kretz R, Bauer S, et al. Prospectively assessed changes in lamotrigine-concentration in women with epilepsy during pregnancy, lactation and the neonatal period. Epilepsy Res **2009**; 85(1): 60-4.

213. Tran TA, Leppik IE, Blesi K, Sathanandan ST, Remmel R. Lamotrigine clearance during pregnancy. Neurology **2002**; 59(2): 251-5.

214. Stader F, Siccardi M, Battegay M, Kinvig H, Penny MA, Marzolini C. Repository describing an aging population to inform physiologically based pharmacokinetic models considering anatomical, physiological, and biological age-dependent changes. Clin Pharmacokinet **2019**; 58(4): 483-501.

215. DuBOIS D, DuBOIS EF. Fifth paper the measurement of the surface area of man. Archives of Internal Medicine **1915**; XV(5_2): 868-81.

216. Gaohua L, Abduljalil K, Jamei M, Johnson TN, Rostami-Hodjegan A. A pregnancy physiologically based pharmacokinetic (p-PBPK) model for disposition of drugs metabolized by cyp1a2, cyp2d6 and cyp3a4. Br J Clin Pharmacol **2012**; 74(5): 873-85.

217. Osiyemi O, Yasin S, Zorrilla C, et al. Pharmacokinetics, antiviral activity, and safety of rilpivirine in pregnant women with HIV-1 infection: Results of a phase 3b, multicenter, open-label study. Infect Dis Ther **2018**; 7(1): 147-59.

218. Zhang H, Hindman JT, Lin L, et al. A study of the pharmacokinetics, safety, and efficacy of bictegravir/emtricitabine/tenofovir alafenamide in virologically suppressed pregnant women with HIV. Aids **2024**; 38(1): F1-f9.

219. Kathleen M. Powis, Mauricio Pinilla, Lauren Bergam, et al. Pharmacokinetics and virologic outcomes of bictegravir in pregnancy and postpartum. Conference on Retroviruses and Opportunistic Infections. Seattle, Washington, US, **2023**.
